# Targeting Ovarian Carcinoma with TSP-1:CD47 Antagonist TAX2 Activates Anti-Tumor Immunity

**DOI:** 10.3390/cancers13195019

**Published:** 2021-10-07

**Authors:** Albin Jeanne, Thomas Sarazin, Magalie Charlé, Catherine Moali, Caroline Fichel, Camille Boulagnon-Rombi, Maïté Callewaert, Marie-Christine Andry, Eric Diesis, Frédéric Delolme, Damien Rioult, Stéphane Dedieu

**Affiliations:** 1 SATT Nord, 25 Avenue Charles Saint-Venant, 59800 Lille, France; a.jeanne@apmonia-therapeutics.com (A.J.); magalie.charle1@univ-reims.fr (M.C.); 2 Campus Moulin de la Housse, UFR Sciences Exactes et Naturelles, Université de Reims Champagne-Ardenne, 51100 Reims, France; thomas.sarazin@univ-reims.fr; 3 CNRS UMR 7369, Matrice Extracellulaire et Dynamique Cellulaire, MEDyC, 51 rue Cognacq Jay, 51100 Reims, France; cboulagnon-rombi@chu-reims.fr; 4 Apmonia Therapeutics SAS, CREA, 2 esplanade Roland Garros, 51100 Reims, France; 5 Laboratoire de Biologie Tissulaire et Ingénierie Thérapeutique, LBTI, Univ. Lyon, Université Claude Bernard Lyon 1, CNRS, L UMR 5305, 69367 Lyon, France; catherine.moali@ibcp.fr (C.M.); frederic.delolme@ibcp.fr (F.D.); 6 Laboratoire d’Anatomie Pathologique, Université de Reims Champagne-Ardenne, UFR Médecine, 51100 Reims, France; caroline.fichel@univ-reims.fr; 7 Laboratoire Central d’Anatomie et de Cytologie Pathologique, CHU de Reims, 51100 Reims, France; 8 Institut de Chimie Moléculaire de Reims, ICMR, CNRS UMR 7312, Institut de Chimie Moléculaire de Reims, ICMR, 51100 Reims, France; maite.callewaert@univ-reims.fr (M.C.); marie-christine.andry@univ-reims.fr (M.-C.A.); 9 Protein Science Facility, Univ. Lyon, SFR Biosciences, ENS de Lyon, INSERM US8, CNRS UMS 344, UCBL, 50 Avenue Tony Garnier, 69007 Lyon, France; eric.diesis@ibcp.fr; 10 Campus Moulin de la Housse, Plateau Technique Mobile en Cytométrie Environnementale MOBICYTE, UFR Sciences Exactes et Naturelles, Université de Reims Champagne-Ardenne/INERIS, 51000 Reims, France; damien.rioult@univ-reims.fr

**Keywords:** ovarian cancer, TSP-1, CD47, peptide, immunotherapy

## Abstract

**Simple Summary:**

Due to the nonspecific nature of disease symptoms and late diagnosis, prognosis for ovarian cancer remains poor, while its incidence is increasing dramatically. Current treatment options lead to recurrence for over 80% of patients, and there is a real and urgent need to identify new therapeutic targets, especially in the field of immuno-oncology. Among possibilities, thrombospondin-1 (TSP-1) is a matricellular protein being overexpressed within ovarian tumors, for which interaction with CD47 receptor was reported as directly inhibiting adaptive immunity. We engineered the first-ever orthosteric antagonist that is selective for TSP-1:CD47 interaction, namely TAX2. TAX2 is a cyclic peptide targeting tumor-overexpressed thrombospondin-1 (TSP-1) to prevent CD47 receptor activation. TAX2 acts as a modulator of the tumor-tolerant microenvironment, reprogramming highly vascularized tumors into poorly angiogenic ones, while concomitantly activating the tumor-inhibiting immune system. A large body of in vivo efficacy data support the proof-of-concept for TAX2 use as an anti-cancer therapy.

**Abstract:**

TAX2 peptide is a cyclic peptide that acts as an orthosteric antagonist for thrombospondin-1 (TSP-1) interaction with CD47. TAX2 was first described for its anti-angiogenic activities and showed anti-cancer efficacy in numerous preclinical models. Here, we aimed at providing an extensive molecular characterization of TAX2 mode of action, while evaluating its potential in ovarian cancer therapy. Multidisciplinary approaches were used to qualify a TAX2 drug candidate in terms of stability, solubility and potency. Then, efficacy studies, together with benchmark experiments, were performed in relevant mouse models of ovarian carcinoma. TAX2 peptide appears to be stable and soluble in clinically relevant solvents, while displaying a favorable safety profile. Moreover, clinical data mining allowed for the identification of TSP-1 as a relevant pharmacological target in ovarian cancer. In mice, TAX2 therapy inhibits ovarian tumor growth and metastatic dissemination, while activating anti-cancer adaptive immunity. Interestingly, TAX2 also synergizes when administered in combination with anti-PD-1 immune checkpoint inhibitiors. Altogether, our data expose TAX2 as an optimized candidate with advanced preclinical characterization. Using relevant syngeneic ovarian carcinoma models, we highlighted TAX2’s ability to convert poorly immunogenic tumors into ones displaying effective anti-tumor T-cell immunity.

## 1. Introduction

The extracellular matrix (ECM) constitutes a highly complex network of secreted macromolecules that provides a structural and functional dynamic scaffolding, thereby orchestrating the interplay between cells within the microenvironment. It is now recognized that targeting appropriate components of the tumor microenvironment may be of particular interest in developing innovative anti-tumor therapeutic approaches. Thrombospondin-1 (TSP-1) is a 450 kDa homotrimeric glycoprotein acknowledged as a key matricellular protein within the tumor microenvironment [1,2,3]. Due to its multi-modular organization, TSP-1 can bind a wide variety of ligands, including other ECM components, extracellular proteases, growth factors and cell-surface receptors, such as LRP-1, integrins, CD36 and CD47 [4,5]. It is thus not surprising that TSP-1 exhibits pleiotropic effects during tumor development and progression, not only by regulating tumor cell behavior, but also by impacting stromal cells that reside within a tumor microenvironment. Studies conducted over many years have attributed to TSP-1 features that may somehow appear contradictory [5,6]. This has not prevented the development of TSP1-derived agents. To provide a better understanding of TSP-1 overall contribution to malignancies, TSP-1 concentrations at local sites, as well as its origin and temporality of its secretion, are major criteria to be considered.

CD47 activation by TSP-1 is emerging as a critical signaling axis in driving tumor behavior, as it is indeed involved in the regulation of tumor cell viability, differentiation of cancer stem cells, tumor angiogenesis and vessel perfusion, and resistance to chemo- and radiotherapy [1,7,8], as well as inhibition of anti-tumor immune response. TSP-1 binding to CD47 was indeed found to directly control adaptive immunity by inhibiting T-cell differentiation, activation and proliferation [9,10,11], while reducing macrophage activation [12]. Strategies aiming at blocking the TSP-1/CD47 axis are currently in the limelight of innovations for the management of cancer. To date, most efforts focused on targeting CD47 by using blocking antibodies, especially in the purpose of disrupting CD47/SIRPα signaling to promote cancer-cell phagocytosis. Preclinical efficacy has been demonstrated in diverse models [13], and several clinical trials are currently underway. However, CD47 is a ubiquitously expressed receptor with particular high expression levels in platelets, endothelial and red blood cells. Thus, general targeting of CD47 by using antibody strategy is likely to induce important adverse events, such as phagocytic-induced severe anemia, thrombocytopenia and splenomegaly, as previously reported during non-clinical work [14,15], as well as in Phase I clinical trials [16]. Furthermore, recent data reported resistance under CD47 blockade both to phagocytosis in melanoma [17] and to chemotherapy-induced senescence [18], highlighting additional obstacles for an anti-CD47 strategy.

For these reasons and because TSP-1 is found to be overexpressed in a wide range of solid tumors [19,20,21], we opted for a selective targeting of TSP-1 rather than CD47. We recently engineered the first-ever orthosteric antagonist being selective for TSP-1:CD47 interaction, namely TAX2. TAX2 is a cyclic 12 amino-acids peptide derived from CD47, that was demonstrated to bind the C-terminal domain of TSP-1 to selectively disrupt TSP-1 binding to CD47 [22]. In syngeneic melanoma models, TAX2 treatment disrupts tumor vascularization, induces extensive tumor necrosis [22] and inhibits the formation and growth of lung metastases [23], while promoting intra-tumor lymphocytic infiltration [24]. TAX2 administration also inhibits growth and vascularization of human pancreatic carcinoma tumors [22] while it induces tumor regression in mice engrafted with large pre-established neuroblastoma tumors [25]. Recently, TAX2 efficacy was also evidenced in glioblastoma models following PDX intracranial implantation. TAX2 indeed reduces vascular density of orthotopic tumors and inhibits contralateral cell infiltration with promising synergies when used as a combination therapy together with bevacizumab [21]. Thanks to its unique mode of action, TAX2 does not recapitulate the hematologic toxicities related to CD47 blockade [26], hence reaching the full potential of targeting this axis for cancer therapy.

In view of a translational approach, we here document TAX2 stability and solubility to demonstrate that TAX2 overcomes many of the usual limits and concerns associated with peptide-based therapeutic strategies. Furthermore, we highlighted that TSP-1-targeting TAX2 peptide exhibits a unique added value for targeting ovarian cancer (OC) by inhibiting tumor growth, as well as peritoneal dissemination. In addition, our work further stresses TAX2 efficacy in activating an efficient anti-tumor immune response and suggests that one should consider TAX2 peptide to be relevant in designing optimized combination therapy together with other immune checkpoint inhibitors.

## 2. Results

### 2.1. Preparation and Characterization of TSP-1-Targeting TAX2 Peptide

**Figure 1:**
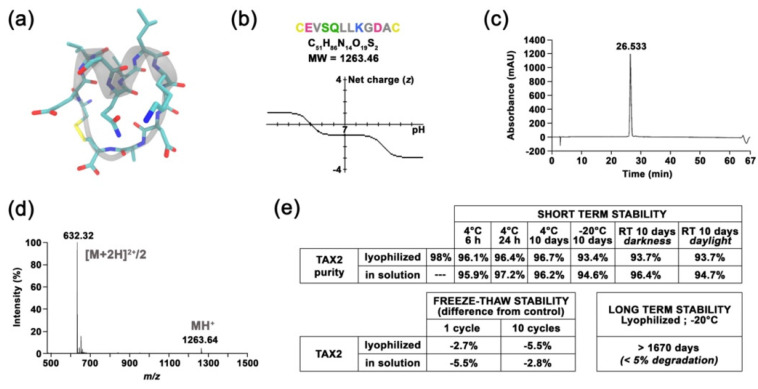

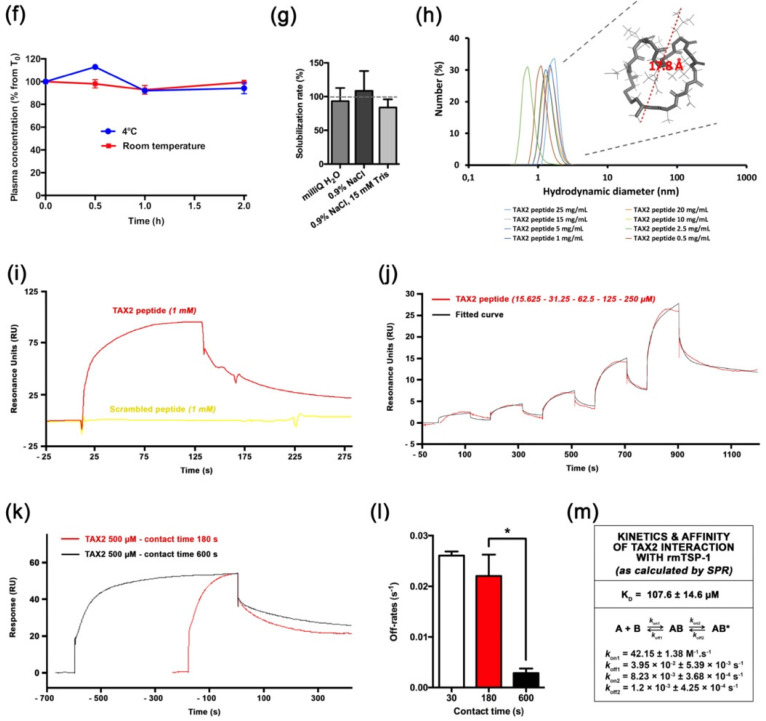
Molecular characterization of TAX2 drug candidate. (**a**) Molecular representation of TAX2 peptide, showing the helical folding of the “core” SQLLKG active sequence (gray), together with a.a. lateral chains (green, red and blue) and the C-C disulphide bridge (yellow), as visualized by using VMD 1.9.3 software. (**b**) TAX2 peptide physicochemical properties. Within TAX2 peptide sequence (CEVSQLLKGDAC), cysteines are indicated in yellow, acidic residues in pink, aliphatic residues in gray, polar residues in green and basic residue in blue. TAX2 chemical formula and molecular weight are indicated, while graph shows the peptide’s net charge vs. pH, as obtained from PepCalc online tool (www.pepcalc.com; Innovagen AB; accessed on 22 February 2018). (**c**) TAX2 peptide quality control: Analytical C18 reversed-phase HPLC profile (λ = 215 nm) of TAX2, indicating the homogeneity and purity of the peptide synthesized by Fmoc chemistry. (**d**) ESI–MS spectrum of double-charged TAX2 peptide (positive ion mode; parent ion *m/z* = 632.32). (**e**) Stability assays: several storage conditions, as well as handling procedures, were applied to TAX2 peptide (lyophilized and/or reconstituted in H2O solution) prior to RP-HPLC analysis, and obtained purity results are reported in the tables. Product and solution appearances were also controlled, while ESI–MS analysis revealed that reference spectra remain unchanged between all tested parameters according to CPTAC assay characterization guidelines (https://assays.cancer.gov). (**f**) Evaluation of TAX2 peptide stability in rat plasma: 500 µL of freshly collected EDTA plasma from Sprague Dawley rats was spiked with TAX2 peptide at the nominal concentration of 50 ng · mL^−1^ and incubated for 2 h at either 4 °C (blue) or room temperature (red). Plasma concentration was evaluated every 30 min through HPLC–MS/MS detection of remaining TAX2 (*n* = 2 per condition). Results are expressed as a percentage from concentration at T0. (**g**) TAX2 solubility assay: TAX2 peptide was solubilized at incremental doses (0.25 to 20 mg · mL^−1^) in either MilliQ water, 0.9% NaCl or 0.9% NaCl buffered with 15 mM Tris. Sample absorbance at 205 nm was measured by using a NanoVue PlusTM spectrometer (path length: 0.05 cm). TAX2 peptide molar absorptivity (extinction coefficient) at 205 nm was determined according to Reference [27], so as to calculate TAX2 concentrations from several measurements within a linear range for absorbance vs. concentration. Calculated concentrations were then compared to powder weighing (corrected for peptide purity and content) to estimate solubilization rates. (**h**) Size (hydrodynamic diameter) distribution (in number %) obtained for TAX2 peptide solutions extemporaneously prepared in 0.9% NaCl at different concentrations (0.5, 1,2.5, 5, 10, 15, 20 and 25 mg · mL^−1^). Particle size distribution was assessed by dynamic light-scattering measurements and analyzed by using Malvern Zetasizer v7.13. Insert displays TAX2 peptide monomer as modeled by using PyMOL molecular graphics system v.2.1.1 (Schrödinger LLC, New York, NY, USA), with a calculated largest dimension of 17.8 Å. (**i**–**l**) Surface plasmon resonance binding analyses. For each figure, one representative experiment out of three is shown. (**i**) Interaction of TAX2 (red) or a scrambled peptide (yellow, both at 1 mM) injected over immobilized rmTSP-1 (2931 RU), at a flow rate of 30 µL.min-1 for 120 s. (**j**) Concentration dependence of binding of TAX2 peptide binding (red; 15.625, 31.25, 62.5, 125 and 250 µM) to immobilized rmTSP-1, as determined by using a single-cycle kinetic analysis experiment (i.e., samples are injected sequentially in the same cycle with no regeneration between sample injections). The black line displays the best fit obtained by using the Biacore T200 evaluation software v3.1 (GE Healthcare Limited, Chicago, IL, USA) (“two-state reaction” model), which was also used for the determination of kinetic parameters. (**k**,**l**) A contact time experiment to confirm a two-state binding model for TAX2:TSP-1 interaction. Adjusted binding curves for TAX2 peptide on immobilized rmTSP-1 with two different exposure times of 180 s (red) and 600 s (black) are shown (**k**). Alignment of the curves to analyte injection end point shows a decreased dissociation rate after the 600 s injection, revealing a stabilizing conformational change. (**l**) Histogram displays dissociation rates measured from three independent experiments at 30, 180 and 600 s (mean ± SEM, *t*-test, * *p* < 0.05). (**m**) Kinetic parameters for the TAX2:TSP-1 interaction obtained with the two-state reaction model. Mean ± SEM values of K_D_, k_on_ and k_off_ values calculated from three similar experiments are provided in the table.

TAX2 drug candidate was engineered as a disulfide linked 12 amino-acid cyclic peptide stretch of CD47 (Figure 1a), which is negatively charged at physiological pH (Figure 1b). Following Fmoc/tBu solid-phase synthesis and then peptide purification, a quality control was systematically performed as follows: reversed-phase HPLC illustrates peptide purity to be >98% (Figure 1c), while mass spectrometry verifies peptide composition (Figure 1d). Interestingly, neither peptide storage conditions and handling, nor repeated freeze–thaw cycles and light exposure impact TAX2 peptide stability (Figure 1e). In addition, no ring-opening was detectable through ESI-MS analysis, while the peptide also remains stable in freshly collected plasma up to 2 hours (Figure 1f). TAX2 is completely soluble in aqueous solvents (Figure 1g), and A_205_ measurements found normal saline (0.9% NaCl) to be a suitable vehicle. Knowledge on size, shape and morphology of the particles is of great importance in developing pharmaceutical formulations, especially for therapeutic peptides. Indeed, solubility of TAX2 in 0.9% NaCl was also confirmed by dynamic light scattering within the 0.5 to 25 mg · mL^−1^ concentration range (Figure 1h).

### 2.2. TAX2 Peptide Binds TSP-1 Specifically

We used a surface plasmon resonance (SPR) approach to investigate TAX2 interaction with recombinant mouse TSP-1. Indeed, sequence alignments showed that TAX2 target sequence on TSP-1 C-terminal domain (1034-RFYVVMWK-1041) is a conserved property of all vertebrate TSP-1 sequences [26]. Biacore experiments showed that rmTSP-1 binds the TAX2 peptide in a specific manner, as it gives rise to a clear SPR signal when injected at 1mM while a scrambled peptide does not elicit any response (Figure 1i,j). Previous in silico simulations highlighted that TAX2 interaction with TSP-1 C-terminal domain implies the opening of a hydrophobic pocket through the disruption of an electrostatic scratch on the protein [22,28], suggesting that a conformational change could occur upon TAX2 binding. Accordingly, variations of contact time (30 s, 3 min and 10 min) were performed in a separate SPR experiment with 500 µM TAX2 to confirm the occurrence of so-called “linked reactions” (Figure 1k,l). A statistically significant difference (*, *p* < 0.05) in the dissociation rates calculated from three independent experiments was observed between 180 vs. 600 s contact time (Figure 1l), thereby confirming the conformational change of the TAX2:TSP-1 complex over time towards a more stable complex. Accordingly, experimental binding data were best-fitted, using the “two-state” binding model (Figure 1j), and corresponding apparent dissociation constant and kinetic values are summarized in Figure 1m. Of note, a previous microscale thermophoresis study has reported a binding affinity estimated in the low micromolar range [23]. However, one should not directly compare data obtained by using distinct TSP-1 (purified from human vs. recombinant mouse isoform), as well as different solubilization buffers. 

### 2.3. TSP-1 and CD47 Expression Correlates with Poor Outcome in Ovarian Carcinoma

**Figure 2:**
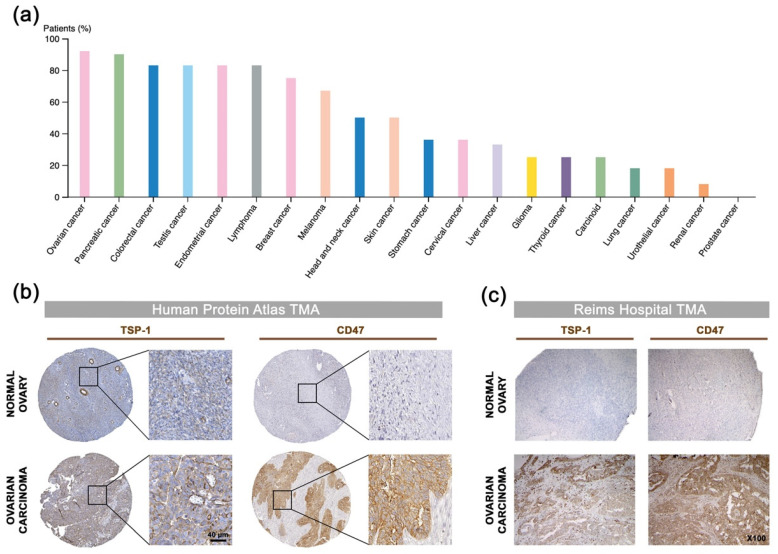

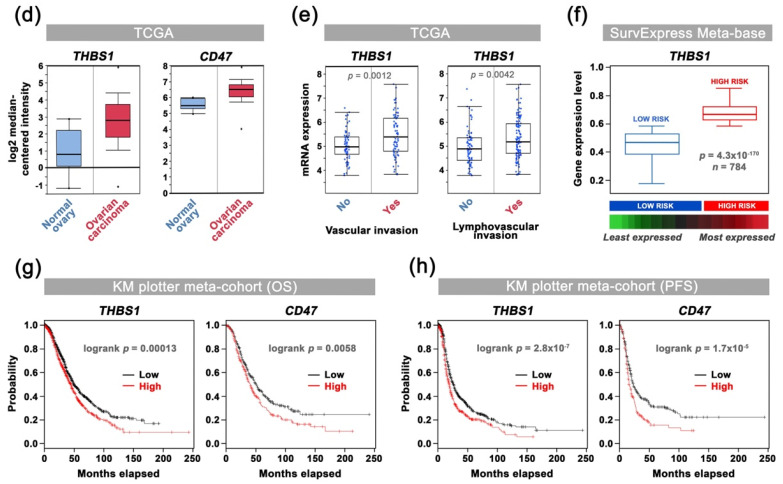
Correlation of TSP-1 and CD47 expression with patient outcome in ovarian carcinoma: a comprehensive review of clinical data. (**a**) Analysis of TSP-1 protein expression among 20 cancer types. The histogram displays the percentage of patients being positive for TSP-1 by IHC (validated antibody: CAB033678) for each cancer type, as retrieved among cohorts from the Human Protein Atlas (available from www.proteinatlas.org; accessed on 11 January 2018). (**b**,**c**) Microphotographs of TSP-1 and CD47 IHC in normal ovary and ovarian carcinoma tissue microarrays from (**b**) the Human Protein Atlas dataset, as well as from (**c**) a Reims Hospital cohort. (**d**) Results for *THBS1* (probe: 201110_s_at) and *CD47* (213857_s_at) differential gene expression analysis (log2, median-centered; Affymetrix U133 signal) among TCGA dataset, as retrieved by using the Oncomine^TM^ platform. Boxes show the median and interquartile range, while bottom and top bars of the whisker indicate the 10^th^ and 90^th^ percentiles, respectively. Min/max values are also indicated (bullet) and more details are indicated in Supplementary Materials Table S1. (**e**) Plots of *THBS1* gene expression (log2; Affymetrix U133 signal) vs. vascular invasion, as well as lymphovascular invasion clinical attributes, as retrieved among TCGA ovarian carcinoma cases by using only the cBio Portal. Student’s *t*-test *p*-values are indicated. (**f**) Box-plot shows *THBS1* expression across risk groups from publicly available OC metabase (SurvExpress tool), including the *p*-value testing for difference, using *t*-test. Heat map displays *THBS1* expression in risk groups, with low expression being represented in green grades and high expression in red grades. Calculated *β* coefficient from the Cox fitting (i.e., linear relationship between gene expression and prognostic index) was 0.908 for *THBS1* gene (with associated *p*-value = 0.016). (**g**,**h**) Kaplan–Meier analysis for (**g**) overall (*n* = 1656) and (**h**) progression-free (*n* = 1435) survival rates of OC patients (KM-plotter dataset) grouped by *THBS1* or *CD47* expression. Logrank test *p*-value is indicated.

Thrombospondin-1 (TSP-1) was mentioned for the first time two decades ago as a potential druggable biological target in OC when elevated *THBS1* mRNA levels were found within ovarian tumors, when compared to borderline epithelial specimen [29]. Pan-cancer analysis of TSP-1 protein expression was performed summarizing data obtained from the Human Protein Atlas [30], hereby revealing that TSP-1 expression seems extremely relevant in the context of OC with over 90% of OC patients having subgroups of tumor cells with positive TSP-1 staining (Figure 2a,b). IHC analysis among tissue samples from a Reims hospital cohort further confirms that both TSP-1 and CD47 were found to be overexpressed by cancer cells, as well as tumor-infiltrating mononuclear inflammatory cells in ovarian carcinoma (Figure 2c), thereby corroborating the pattern observed in the Human Protein Atlas web portal (Figure 2b), as well as recent reports on CD47 expression in epithelial OC [31]. Consistently, differential gene expression among TCGA showed a trend for both TSP-1 and CD47-encoding genes (i.e., *THBS1* and *CD47*, respectively) to be upregulated in ovarian carcinoma patients (Figure 2d), especially with elevated *THBS1* mRNA expression in case of vascular and lymphovascular invasion (Figure 2e). *THBS1* mRNA expression levels also significantly correlate with poor prognosis for ovarian carcinoma patients (Figure 2f), while both *THBS1* and *CD47* expression are associated with being decreased overall, as well as progression free survival (Figure 2g,h). Altogether, this round of data arising from multiple clinical data analyses establishes the relevance of targeting the TSP-1:CD47 axis in ovarian carcinoma.

### 2.4. TAX2 Peptide Inhibits Tumor Growth of Human Ovarian Carcinoma Xenografts

**Figure 3:**
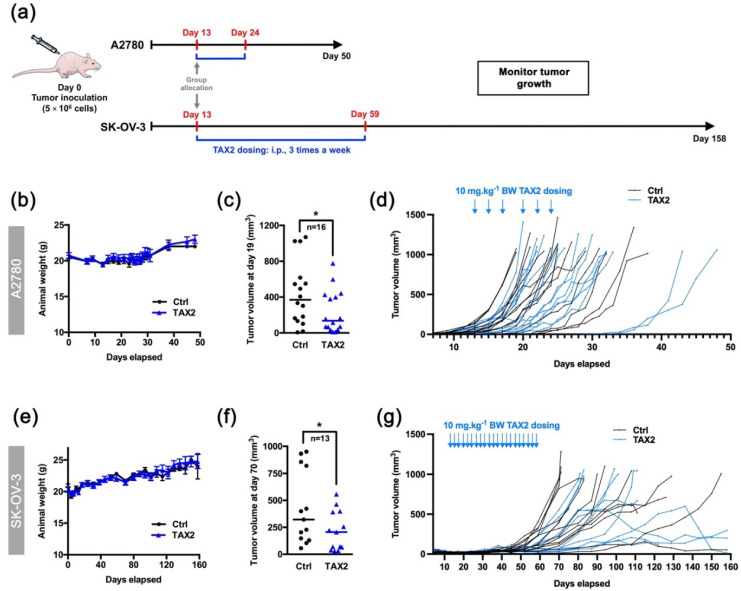
Therapeutic evaluation of TAX2 peptide in the A2780 and SK-OV-3 xenograft models. (**a**) 5 × 10^6^ A2780 or SK-OV-3 human ovarian carcinoma cells were s.c. implanted in BALB/c nude mice, and then i.p. administrations of either vehicle (0.9% NaCl) or TAX2 peptide (10 mg · kg^−1^ BW) were performed 3 times a week for 2 weeks (A2780 model: **b** to **d** or 7 weeks (SK-OV-3 model: **e** to **g**) (*n* = 18–22 per group). Before applying first administration on day 13, we ensure that mean tumor volumes between groups were comparable. At treatment administration starting point, note that subcutaneous xenografts had already established tumor vasculature as controlled by micro-computed tomography. Mice were regularly checked for BW, tumor volume and clinical signs. (**b**,**e**) Evolution of mice body weights (mean ± SEM). (**c**, **f**) Scatter dot plots of individual calculated tumor volumes before the first animals reached endpoint for euthanasia (>1 cm^3^), i.e., on day 19 (**c**: A2780 model) and on day 70 (**f**: SK-OV-3 model), only considering mice having a palpable and measurable tumor at said time-points. Line, median (Mann–Whitney *U* test, * *p* < 0.05). (**d**,**g**) Evolution of individual animal tumor volumes in mm^3^.

Thanks to interspecies conservation of TAX2 target sequence on TSP-1 C-terminal domain [26], engraftment of human tumor cells in mouse models is relevant in the purpose of investigating TAX2 peptide effects on tumor growth. Indeed, TAX2 anti-cancer properties have been extensively documented including various therapeutic set-ups in xenograft models of pancreatic carcinoma [22], melanoma [23], neuroblastoma [25] and glioblastoma [21]. Here, A2780 or SK-OV-3 human ovarian carcinoma cells with distinct aggressiveness properties were subcutaneously xenotransplanted to BALB/C nude mice, which were later randomized according to tumor volume distribution prior to systemic administration of TAX2 peptide (Figure 3a). While TAX2 administration thrice a week appeared to be safe in these models (Figure 3b,e), a 2-fold inhibition of tumor growth was observed in TAX2-treated animals at early time-points (Figure 3c,f). It should also be noted that comparable treatment-related effects are observed in two mouse models exhibiting different degrees of tumor aggressiveness (Figure 3d,g). Such results are consistent with the considerable amount of work performed over the last years to elucidate TAX2 mode of action, leading to the conclusion that the anti-cancer benefits of TAX2 therapy in xenograft models to rely on the peptide anti-angiogenic properties [21,22,23,24,25]. Besides, another crucial role of the TSP-1:CD47 signaling axis emerged that leads to tolerogenic signals allowing tumor immune escape through direct inhibition of T-cell activation [10,32]. Hence, we then considered immunocompetent host animals to investigate the putative effects of TAX2 peptide on the modulation of anti-tumor immune responses.

### 2.5. TAX2 Therapy Activates Anti-Tumor Immunity in Ovarian Carcinoma Models

**Figure 4:**
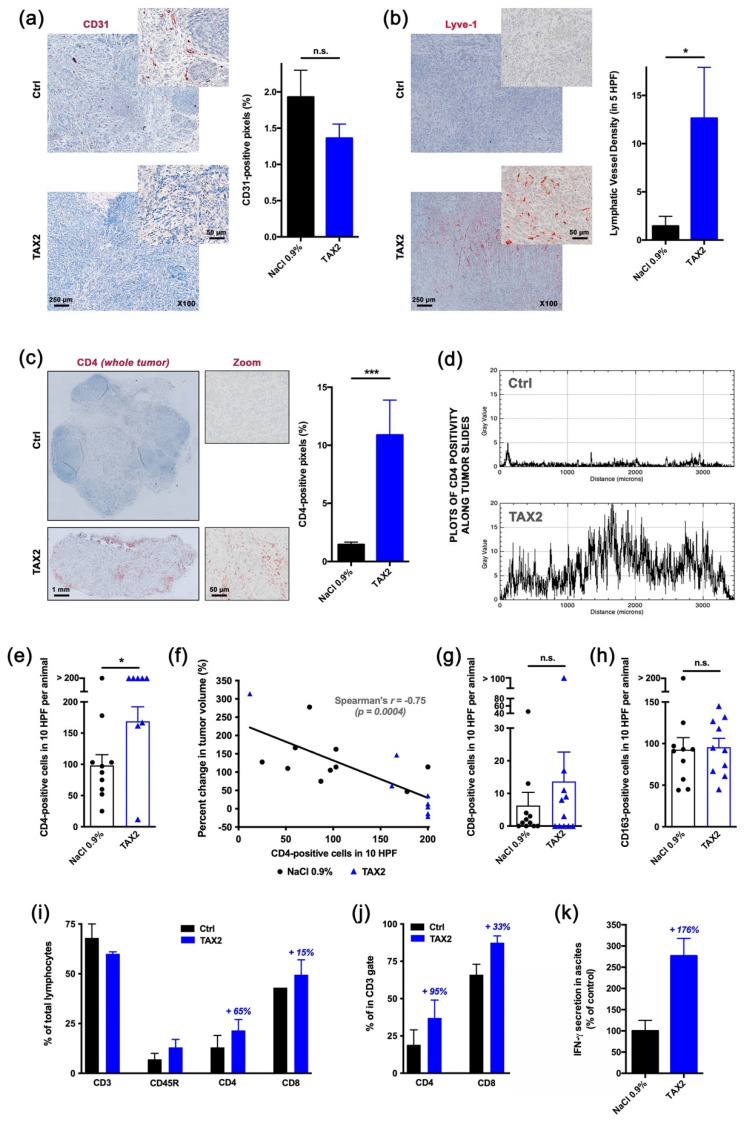
Proof-of-mechanism study for TAX2 use as an anti-tumor immunomodulatory drug. The effects of TAX2 peptide treatment on the activation of an anti-tumor immune response were investigated in two syngeneic models of epithelial ovarian carcinoma. We inoculated 5 × 10^6^ ID8 cells either subcutaneously or intraperitoneally to C57BL/6JRj mice in order to mimic primary tumor growth (**a**–**h**) and metastatic dissemination (peritoneal carcinosis, **i**–**l**), respectively. TAX2 treatments (30 mg · kg^−1^ BW) vs. vehicle injections (0.9% NaCl) were performed thrice weekly for 4 and 8 weeks for the s.c. and i.p. ID8 models, respectively (*n* = 11 per group for both models). (**a**–**h**) IHC staining of s.c. ovarian carcinoma allografts for the analysis of the vascular/lymphovascular features, as well as immune-cell-infiltration profile. (**a**) Representative microphotograph for CD31 immunostaining of intra-tumor vascular structures. Histogram displays results of automated quantification for the percent of CD31-positive pixels across the whole tumor section (mean ± SEM, Mann–Whitney U test; n.s., not significant). (**b**) Lymphatic vessel density analysis. Microscopic views are shown, while histogram displays the number of Lyve-1-positive functional lymphatic vessels in 5 high power fields (HPF), as determined by a pathologist who was blinded to the treatment (mean ± SEM, Mann–Whitney U test, * *p* < 0.05). (**c**–**f**) Macroscopic views (×20 magnification) of s.c. tumor allograft sections after CD4 immunostaining (**c**, left panel). Histogram (**c**, right panel) displays results of automated quantification for the percent of CD4-positive pixels across the whole tumor section (mean ± SEM, Mann–Whitney U test, *** *p* < 0.001), while distribution of CD4 positivity along the tumor’s largest dimension (**d**) is displayed as gray values plotted against distance (in µm). For IHC, stainings that did not match quality control were blindly excluded by a pathologist. To confirm the specificity of CD4 immunostaining for tumor-infiltrating lymphocytes (TiLs), CD4-positive cells were counted in 10 HPF per animal (**e**, mean ± SEM, Mann–Whitney U test, * *p* < 0.05), and then the correlation between the number of CD4-positive infiltrating TiLs and the percent change in tumor volume was established (**f**). Linear regression was performed (black line) and *r* coefficient arising from non-parametric two-tailed Spearman test was determined (*p* = 0.0004). (**g**,**h**) CD8-positive cytotoxic T cells (**g**), as well as CD163-positive macrophages (**h**), were also counted in 10 HPF per animal (histograms display mean ± SEM, Mann–Whitney U test; n.s., not significant). (**i**–**k**) Phenotypic analysis of peritoneal immune cells by flow cytometry. Upon sacrifice, ascites was removed from ID8 tumor bearing mice and spun at 300 g for 10 min to isolate the cellular component. A peritoneal lavage was performed to obtain resident cells from mice presenting no abdominal distension. Cells were stained for CD3, CD45R, CD4 and CD8 positivity and then analyzed by flow cytometry. (**i**) The percentage of total lymphocytes being positive for each marker was calculated and displayed as mean ± SEM. (**j**) Proportions of CD4^+^ and CD8^+^ cells were also computed among the CD3^+^ T-cell population. (**k**) IFN-γ production in the non-cellular fraction of ascitic fluid or peritoneal lavages was assessed by ELISA and displayed as percent of control group (mean ± SEM).

To further decipher TAX2 peptide immune-oncology (IO) mechanism of action, we considered the ID8 murine ovarian carcinoma, a commonly accepted syngeneic model that recapitulates human high-grade epithelial OC [33,34]. Analysis of ID8 s.c. tumors from TAX2-treated animals for microvascular density (CD31 IHC) indicated that TAX2 anti-tumor effects do not rely here on its anti-angiogenic properties (Figure 4a). Interestingly, a significant increase in lymphatic vessels perfusing ID8 tumors was observed under TAX2 treatment (Figure 4b). Of note, lymphatic vessels are required for initiation of anti-tumor immunity and correlated to immune cells infiltration in metastatic cancers [35]. Analysis of ID8 s.c. tumors immune profile revealed that TAX2 treatment not only increases the number of infiltrating CD4+ (Figure 4c,e), but also stimulates deeper T-cell infiltration within the whole tumor sections (Figure 4d). Such increase in T-cell infiltration correlates with tumor growth inhibition (Figure 4f). While a trend towards increased CD8+ cell recruitement was observed under TAX2 treatment (Figure 4g), TAX2 did not impact macrophages tumor recruitment from peritumoral areas (Figure 4h), thereby demonstrating that TAX2 effects are mostly focused on adaptive immunity modulation. TAX2 peptide immunostimulatory properties were also confirmed in a metastatic model that mimics ID8 ovarian carcinoma peritoneal dissemination, in which flow cytometry analysis of ascites fluids indicated a tendency of increased recruitment of CD4+ T cells in TAX2-treated group (Figure 4i,j), while CD45R+ B lineage does not appear to be affected (Figure 4j). Consistent with reports highlighting a role for the pro-inflammatory cytokine IFN-γ in promoting anti-tumor responses through stimulation of the immune system [36], treatment-related cellular effects were accompanied by an increase in IFN-γ concentration within ascitic peritoneal lavages from TAX2-treated mice (Figure 4k).

### 2.6. TAX2 Treatment Synergizes with Immune Checkpoint Inhibition

**Figure 5:**
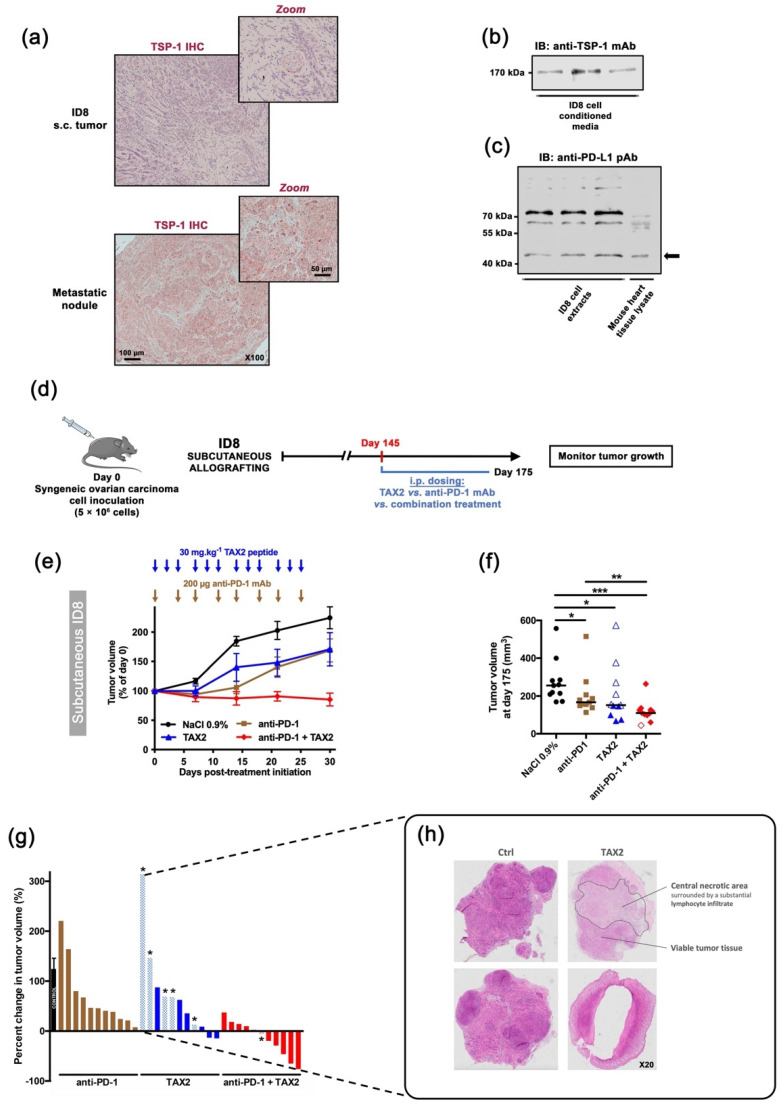
Therapeutic evaluation in subcutaneous syngeneic ID8 ovarian carcinoma allografts: TAX2 monotherapy and combination with anti-PD-1 mAb. (**a–c**) Characterization of the ID8 mouse ovarian epithelial cell line. (**a**) ID8 inoculation in C57BL/6 mice may result in s.c. (left panel) or peritoneal (right panel) tumor formation, in both of which ID8 tumor cells express TSP-1 (i.e., TAX2 peptide direct molecular target) in large amount, as revealed by immunohistochemistry. (**b**,**c**) Western blot analyses confirmed that ID8 cells (**b**) release TSP-1 in the extracellular medium when maintained in culture, while (**c**) they also express the PD-L1 protein at their cell surface (arrow). The corresponding uncropped immunoblots were presented in Supplementary Materials Figure S1. (**d–g**) 5 × 10^6^ ID8 cells were s.c. implanted in C57BL/6JRj mice in order to recapitulate subcutaneous tumor formation (**d**). On day 145 (i.e., when mean tumor volume reached 100–200 mm^3^), mice were randomized (n = 11 per group) and then received the following treatments i.p.: 0.9% NaCl (100 µL, thrice weekly, black circles), TAX2 peptide (30 mg · kg^−1^ mouse weight, thrice weekly, blue triangles), anti-mouse PD-1 mAb (200 µg, twice weekly, brown squares) and combined TAX2/anti-PD-1 therapy (red diamonds). Treatments lasted 4 weeks, during which tumor growth was monitored. (**e**) Evolution of tumor volume expressed as a percent of calculated tumor volume at treatment initiation (mean ± SEM). Arrows indicate treatment time points. (**f**) Scatter dot plot displays individual tumor volume on day 175 (i.e., 30-day post-treatment initiation), with median indicated as black line (Mann–Whitney U test, * *p* < 0.05, ** *p* < 0.01, *** *p* < 0.001). Within TAX2, as well as combination therapy groups, empty symbols (blue △ or red ◊) stand for animals presenting extensive tumor necrosis and/or tumor shrinkage, for whose tumor volumes calculated as 0.5 × L × W^2^ may therefore be artificially high. (**g**) Histogram displays individual percent changes in tumor volume, calculated as described in f) (with treatment initiation day as a reference). Tumor growth in the control group is indicated as mean ± SEM, while checkerboard pattern bars with an asterisk stand for necrotic and collapsed tumors. Whatever the necrosis observed postmortem, no corrective factor is brought on the values represented by the histograms. (**h**) Microphotographs of HES-stained ID8 subcutaneous tumors, highlighting extensive central tumor necrosis under TAX2 treatment.

**Figure 6:**
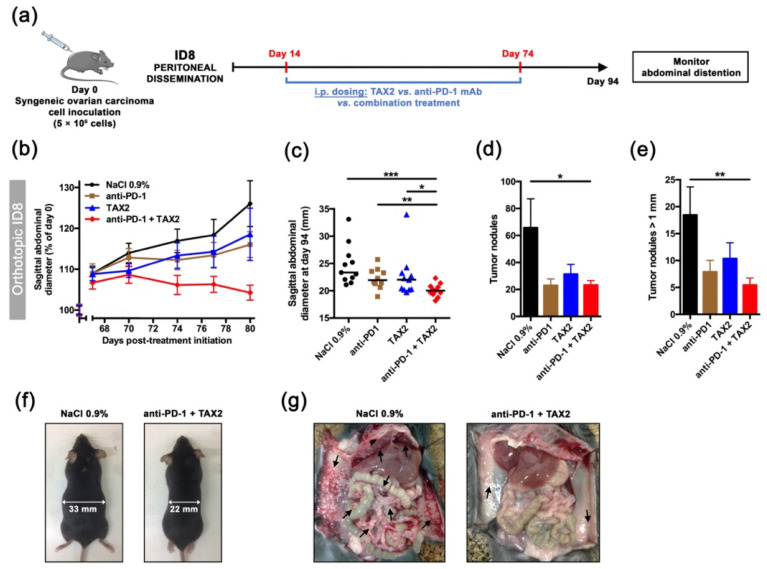
Validation in an orthotopic, syngeneic model of metastatic peritoneal carcinomatosis. (**a**–**g**) 5 × 10^6^ ID8 cells were i.p. implanted in C57BL/6JRj mice in order to recapitulate peritoneal metastatic dissemination (**a**). On day 14, mice were randomized (*n* = 11 per group) and then received the following treatments i.p.: 0.9% NaCl (100 µL, thrice weekly, black circles), TAX2 peptide (30 mg · kg^−1^ mouse weight, thrice weekly, blue triangles), anti-mouse PD-1 mAb (200 µg, twice weekly, brown squares) and combined TAX2/anti-PD-1 therapy (red diamonds). Treatments lasted 8 weeks, during which abdominal distension (as it reflects ascites fluid production) was monitored. (**b**) Evolution of sagittal abdominal diameters, expressed as a percent of measured diameter at treatment initiation (mean ± SEM). (**c**) Scatter dot plot displays individual sagittal abdominal diameters on day 94 (i.e., 80-day post-treatment initiation), with median indicated as black line (Mann–Whitney U test, * *p* < 0.05, ** *p* < 0.01, *** *p* < 0.001). (**f**) Representative photographs of mice at study termination. (**d**-**e**) Histograms show the total number of peritoneal tumor nodules (**d**), as well as tumor nodules with diameter being over 1 mm (**e**), as counted during mice necropsy and expressed as mean ± SEM (Mann–Whitney U test, * *p* < 0.05, ** *p* < 0.01). (**g**) Gross necropsy evaluation for control and combination therapy groups (arrows indicate intraperitoneal tumor masses).

Given the perspective for associating TAX2 with other IO therapies, a combination treatment regimen of TAX2 peptide together with anti-PD-1 mAbs was investigated by using the ID8 ovarian carcinoma model. The rationale for using anti-PD-1 vs. anti-CTLA-4 targeting is based on its wide use and description in the ID8 allograft model [37,38]. Tumorigenic ID8 cells were first ensured for TSP-1 protein expression (Figure 5a,b), while constitutive PD-L1 (i.e., ligand for the immune checkpoint PD-1) was also checked by Western blot (Figure 5c). Two ID8 cells implantation sites were considered, i.e., subcutaneous or peritoneal dissemination, prior treatment with TAX2 peptide and anti-PD-1 mAbs being administered either alone or in combination (Figure 5d). In mice subcutaneously engrafted with ID8 ovarian carcinoma cells, TAX2 inhibited tumor growth in a similar extent than anti-PD-1 therapy, while promoting extensive tumor necrosis and a subsequent tumor shrinkage in about half the mice (Figure 5e–h), hence being consistent with what has been observed from other immunocompetent models in which TAX2 was previously investigated [22]. Despite central necrosis observation in TAX2-treated mice, a longitudinal follow-up of mice, using an X-ray computed-tomography approach, did not reveal any change in tumor vasculature, being consistent with data reported in Figure 4a. Interestingly, TAX2 displays additive effects in combination with anti-PD-1 by enhancing tumor regression (Figure 5g). Similar results were obtained from the peritoneal carcinomatosis model (Figure 6a–f), in which TAX2 peptide in combination with anti-PD-1 mAbs drastically inhibited both ascites fluid production (as revealed by smaller abdominal diameters, Figure 6a–c), as well as metastatic dissemination revealed by the number of peritoneal implants and mesenteric tumor mass being observed during necropsy (Figure 6d–f). Here again, the TAX2 effects were maintained up to 20 days following the last peptide administration.

## 3. Discussion

TAX2 peptide was engineered as the first-ever orthosteric antagonist being selective for TSP-1 interaction with CD47 membrane receptor. Here, we provide a molecular characterization of TAX2 peptide including stability, solubility and SPR binding experiments. Additionally, our findings establish the relevance of targeting TSP-1:CD47 axis in ovarian carcinoma, while positioning TAX2 as an innovative immunotherapeutic approach with demonstrated preclinical efficacy, either when considered as a monotherapy or in combination with immune checkpoint inhibition.

While most OC patients respond to initial first-line therapy (i.e., debulking surgery followed by platinum-based chemotherapy) and enter remission, over 80% of patients will undergo recurrence and develop chemo-resistance. Treatment options for relapsed OC are beginning to diversify from single-agent chemotherapies (i.e., paclitaxel and topotecan) to the angiogenesis inhibitor bevacizumab and PARP inhibitors. A recent therapeutic breakthrough has been provided by PARP inhibitors for treatment or maintenance of BRCA1/2-mutated patients (about 15–20% of patients), and it is now approved for epithelial ovarian, fallopian tube and primary peritoneal cancer, as both treatment and maintenance [39]. The obvious unmet medical needs include products for patients who have advanced/recurrent cancer and those with a disease which is refractory or resistant to standard platinum-based chemotherapies. An elegant study by Pinessi et al. demonstrated that TSP-1 is overexpressed within OC patient-derived xenografts (PDX) that acquired resistance to paclitaxel treatment [20]. Interestingly, this report also highlighted a trend for TSP-1 expression being higher in clear cell ovarian carcinoma, a histotype thought to be particularly immune reactive. Consistently, our data establish TSP-1 as a statistically relevant ovarian carcinoma prognostic marker (Figure 2) and underline the relevance of targeting TSP-1 in ovarian carcinoma patients. While thrombospondin-1 (TSP-1) was mentioned for the first time two decades ago when elevated TSP-1 mRNA levels were found within ovarian tumors [29], most efforts focused on disrupting CD47/SIRPα up to now, in the purpose of promoting cancer cell phagocytosis. However, due to CD47 ubiquitous expression, antibody blockade interferes with important physiological roles of the receptor, therefore promoting adverse events such as anemia, thrombocytopenia and splenomegaly [15]. In contrast with a prevailing view consisting in CD47:SIRPα targeting for cancer cure, a crucial role for TSP-1 has recently emerged in the context of immunomodulation, as TSP-1 binding to CD47 was found to directly control adaptive immunity via inhibiting T-cell differentiation, activation and proliferation [9,10], and reduce macrophage activation [12]. This new set of data challenges the current “CD47 dogma” according to which most CD47 inhibitory effects on anti-tumor immunity rely on SIRPα binding, and considerably increase the relevance of inhibiting TSP-1/CD47 axis in cancer therapy. Indeed, a selective targeting of TSP-1 is expected to promote an overall anti-tumor immune response, without interfering with physiological functions of CD47.

TAX2 peptide was first characterized for its anti-angiogenic properties, using in vitro, ex vivo and in vivo models, demonstrating that TAX2 administrations lead to the disruption of tumor-associated vascular networks, thereby promoting tumor necrosis. Such anti-angiogenic effects are mediated by the disruption of VEGFR2 activation and downstream NO signaling that occurs under TAX2 treatment [22]. Hence, anti-cancer properties of TAX2 drug candidate were demonstrated in various indications involving xenograft models in both BALB/c and NMRI nude mice [22,23,25]. Here, the ability of early TAX2 treatments to inhibit the growth of human ovarian tumor xenografted to athymic mice was confirmed by using the SK-OV-3 and A2780 ovarian carcinoma models (Figure 3). To further confirm these data, a translational model, using large pre-established tumors and a therapeutic setting, should be considered, as previously performed when considering neuroblastoma xenografts [25]. However, as discussed above, another central role of the TSP-1:CD47 signaling axis emerged that leads to tolerogenic signals and allows tumor immune escape through direct inhibition of T-cell activation [10,32], as well as by regulating natural killers and dendritic cells functions [40]. At the same time, several reports pointed out a putative overestimation of the CD47:SIRPα interaction in the interpretation of CD47-mediated modulation of anti-tumor immune response [15]. In light of these latest evidence, the putative effects of TAX2 peptide on the modulation of anti-tumor immune responses were investigated. The first evidence for a TAX2-mediated increase in tumor-infiltrating lymphocytes (TiLs) was obtained from the B16 melanoma model. Disruption of TSP-1:CD47 interaction under TAX2 treatment indeed promotes accumulation of infiltrating CD3+ T cells within s.c. implanted melanoma tumors [24]. Here, we considered the ID8 cell tumors, a syngeneic mouse model being useful for the purpose of studying immunomodulation and its impact on the progression of OC. Particularly, i.p. injection of ID8 ovarian carcinoma cells into normal, immune-intact, syngeneic C57BL/6J mice gave rise to tumor nodules throughout the abdominal cavity similar to those observed in women with advanced Stage III and IV OC [41]. Results obtained from both heterotopic (subcutaneous primary tumors), as well as orthotopic (peritoneal carcinomatosis) ID8 ovarian carcinoma models highlighted that TAX2 treatment inhibits tumor growth and metastatic colonization of the peritoneal cavity, while displaying additive effects when combined with anti-PD-1 mAbs (Figure 5 and Figure 6). Interestingly, these effects do not appear to be attributable to the TAX2 anti-angiogenic properties since the s.c. ID8 tumors appeared weakly vascularized (Figure 4a). Additional studies are needed to substantiate the translational value of our data, including the analysis of a control peptide and irrelevant antibodies in the combination setting. Further evaluation of TAX2 in PDX-bearing mice with humanized immune system will be considered to further confirm these results. Comprehensive IHC analyses revealed that TAX2 peptide immune mode of action mostly relies on adaptive immunity, since it does not only increases the number of infiltrating CD4+ T lymphocytes, but also stimulates deeper T-cell infiltration within the tumor mass. On the contrary, TAX2 treatment did not impact macrophage tumor recruitment, which appears to be consistent with TAX2 molecular mode of action that does not affect CD47 binding to its macrophage counter-receptor SIRPα [22]. Joint angiostatic and immunostimulatory effects of TAX2 peptide depend on its dual mode of action, both relying on CD47 antagonization together with CD36 activation [22]. This is consistent with recent data obtained from the ID8 ovarian carcinoma model in which gene therapy vectors engineered to express 3TSR (i.e., CD36-activating TSP-1 fragment), together with a CD47-binding peptide, were shown to inhibit primary tumor growth and development of secondary lesions [42]. 

Selective targeting of tumor-overexpressed TSP-1 at the CD47-binding site rather than targeting the ubiquitous CD47 membrane receptor explains why TAX2 was found to be safe in rodents [26]. In addition, and as expected, benchmark experiments revealed that TAX2 peptide does not recapitulate adverse events relative to anti-CD47 therapies, especially regarding splenomegaly, hematology, red blood cells count, necrosis, hemoglobin concentration, platelets count and leukocyte count (Supplementary Materials Figure S2). Neither TAX2 monotherapy nor combined to anti-PD-1 mAb had any impact on platelet ability to activate (Supplementary Materials Figure S3). Apart from demonstrating TAX2 efficacy, as well as its lack of toxicity [26], we here demonstrate that the TAX2 HPLC/MS analytical methods, storage, solubility and potency assay (SPR experiments, Figure 1) are under control for further clinical development. 

## 4. Materials and Methods 

### 4.1. TAX2 Peptide Synthesis and Quality Control

TAX2 cyclic peptide (CEVSQLLKGDAC; C-C disulphide bridge; acetate salts), acting as an orthosteric antagonist for CD47:TSP-1 interaction [22], was produced by Genecust (Boynes, France) and is obtained from solid-phase peptide synthesis (SPPS), using Fmoc chemistry [43] and fully automated synthesizers. Purity analyzes were carried out on an HPLC Ultimate 300 system (Thermo Fisher Scientific, Waltham, MA, USA). Briefly, 20 µL of the peptide solutions (1 mg · mL^−1^ in MilliQ water, Merck Millipore, Burlington, MA, USA) were injected at a 0.8 mL.min^−1^ flow rate in a Nucleosil C18 120 A column (250 mm × 4 mm; 5 µm) maintained at 30 °C. The peptide elution was performed with a 60 min gradient ranging from 100% of solvent A (H_2_O/TFA 0.1%) to 100% of solvent B (H_2_O/TFA 0.1%/CH_3_CN 70%), while wavelength detection was set at 215 nm. Mass spectra were then acquired on LTQ Velos device (Thermo Fisher Scientific) operating in FIA. Samples (1 mg · mL^−1^ in MilliQ water) were dissolved 1/2000 in a methanol/water/formic acid 0.1% solution (50/50). Acquisitions were performed in enhanced mode scan on a mass range from *m/z* 400 to *m/z* 1800 for 1 min, and all the spectra were combined.

### 4.2. Stability Assays

In order to assess TAX2 peptide stability, both as a lyophilized powder or in aqueous solution (1 mg · mL^−1^ in MilliQ water), as well as to investigate the putative effects of light exposure, temperature and freeze–thaw cycles on peptide handling and storage, the abovementioned HPLC and MS analytical methods were conducted according to guidelines from the Clinical Proteomic Tumor Analysis Consortium (CPTAC) of the US National Cancer Institute [44]. As plasma stability is also critical in the pipeline of drug development [45], 1 µM TAX2 was incubated at 37 °C for 2 h in human plasma while performing HPLC–MS/MS detection of remaining compound every 30 min, using a validated high throughput assay [46].

### 4.3. Solubility Assays

Absorbance at 205 nm has demonstrated its utility in accurate determination of the concentration of any peptide solution, even when there are no aromatic residues present [27]. For that purpose, molar absorptivity (ε_205_) was calculated from TAX2 peptide amino acid sequence, using dedicated online tool (available at http://spin.niddk.nih.gov/clore, accessed on 14 August 2019). Absorbance values at 205 nm were measured on a NanoVue Plus^TM^ spectrophotometer (GE Healthcare, Chicago, IL, USA) using a quartz cuvette with a 0.05 cm path length. TAX2 peptide dilutions were either performed in water, normal saline or Tris-buffered saline. Absorbance (A_205_) was measured at various dilutions within the 0 to 20 mg · mL^−1^ range, and then corresponding TAX2 peptide concentrations were calculated from absorbance, using the Beer–Lambert law. All calculated concentrations within a linear range for absorbance vs. concentration were averaged and then compared to TAX2 lyophilized powder weighing (corrected for peptide purity and content) to estimate solubilization rates.

### 4.4. Dynamic Light Scattering

Dynamic light scattering (DLS) was used to determine the size of TAX2 peptide particles in solution, as well as their polydispersity, i.e., describing the width of the particle size distribution. TAX2 hydrodynamic diameter was measured by using Zetasizer Nano ZS system (Malvern Instruments, Worcestershire, UK) equipped with a laser source (633 nm wavelength) and operating at a scattering angle of 173°. Measurements were carried out at 25 °C, in disposable cuvettes, using a sample volume of 1 mL at various dilutions within the 0 to 25 mg · mL^−1^ TAX2 peptide concentration range, with each sample being measured in triplicates. Measurements were analyzed by using Zetasizer software v7.13.

### 4.5. SPR Spectroscopy Analysis of TAX2:TSP-1 Interaction

Binding of TAX2 to TSP-1 was monitored with surface plasmon resonance (SPR) spectroscopy, using a Biacore T200 system (GE Healthcare). The His capture kit was used to capture recombinant mouse thrombospondin-1 with a C-terminal 6His tag (#7859-TH; R&D Systems, Minneapolis, MN, USA) on CM5 sensor chips (GE Healthcare) according to the manufacturer’s instructions. Briefly, the anti-histidine antibody was covalently attached to the carboxymethylated dextran layer of the sensorchip through amine coupling prior to the capture of rmTSP-1 (50 µg solubilized in 200 µL of 10 mM HEPES at pH 7.4, and then diluted to 20 µg.mL^−1^ in running buffer composed of 10 mM HEPES, 0.15 M NaCl, 0.05% surfactant P20, 5 mM CaCl_2_; pH 7.4). The control channel (reference) was prepared by using the same procedure, except that TSP-1 solution was replaced by buffer alone. Either TAX2 peptide or a previously validated control scrambled peptide (LSVDESKAQGIL) [22] were injected for 120 s over the immobilized ligand at 25 °C in running buffer with a flow rate of 30 µL.min^−1^. Regeneration of active and control channels was performed by using 2 M NaCl. Five analyte concentrations were considered for kinetic analysis and double referencing (subtraction of the reference response, followed by subtraction of the zero concentration) was used for data analysis. Sensorgrams were then fitted by using the two-state reaction (conformational change) interaction model (Biacore T200 evaluation software v3.1). A “linked reactions” control experiment consisting in injecting TAX2 over TSP-1 for different durations (30–600 s) was also performed to confirm the multiphase binding kinetics of TAX2:TSP-1 interaction and validate the choice of the interaction model [47]. The quality of the fit was judged by the residuals and the reduced χ^2^ value. The apparent equilibrium dissociation constant (K_D_) and interaction kinetic parameters were calculated from three separate experiments.

### 4.6. Animal Care and Ethics Committee Approval

C57BL/6JRj and BALB/c nude mice (CBy.Cg-*Foxn1^nu^*/J) mice were purchased from Janvier Labs (Janviers Labs, Saint-Berthevin, France) and Charles River Laboratories (Charles River Laboratories, L’Arbresle, France), respectively. Animals were fed a standard laboratory diet with water and food *ad libitum* and were kept under constant environmental conditions. All studies were performed in compliance with “The French Animal Welfare Act” and following “The French Board for Animal Experiments”. Experiments were conducted under the approval of the French “Ministère de l’Enseignement Supérieur et de la Recherche” (ethics committees no. C2EA-56) in compliance with the “Directive 2010/63/UE”.

### 4.7. Ovarian Cancer Tissue Microarray

For tissue microarray construction, 20 ovarian carcinoma tissue samples were retrieved from the Pathology Department of the Academic Hospital of Reims (Reims, France). Immunohistochemistry (IHC) analysis was performed on paraffin-embedded tissue, using anti-TSP-1 (#ab1823, Abcam, Cambridge, UK) and anti-CD47 (#ab192827, Abcam) primary antibodies, followed by standard streptavidin–biotin–peroxidase complex method (Novolink Polymer and Rabbit Specific HRP/DAB [ABC] detections systems from Leica Biosystems and Abcam, respectively). Sections were counterstained with hematoxylin/eosin (#RE7107, Leica Biosystems, Danaher Corporation, Washington, WA, USA). Control isotypes (#ab172730 and #ab81216, Abcam) were used to confirm specificity of immunostaining, while paired para-carcinoma tissues were used as non-tumor control samples. Microarrays were reviewed by a pathologist (blinded to clinical information) for evaluation of intensity of positive staining in order to examine the expression of TSP-1 and CD47, as well as the protein cellular/subcellular localization. Apart from Reims Hospital tissue microarray, the pattern of TSP-1 staining in normal ovary and ovarian carcinoma tissue was also characterized in the Human Protein Atlas web portal (available from www.proteinatlas.org, accessed on 11 January 2018) [30].

### 4.8. Bioinformatics and Clinical Data Mining

TSP-1 encoding gene (*THBS1*) and *CD47* were assessed for mRNA expression in OC among dataset from The Cancer Genome Atlas (TCGA; https://tcga-data.nci.nih.gov/tcga, accessed on 13 December 2017). The Oncomine^TM^ platform [48] was used to retrieve and analyze data. Differential gene-expression analyses between normal ovary and ovarian carcinoma among the TCGA dataset were based on the log2 median-centered intensity values of appropriate probes for each considered gene. The cBio Cancer Genomics Portal [49,50] was used to establish correlation with corresponding patient clinical data when available. Among previously edited OC meta-base (*n* = 755), risk estimation was performed by using SurvExpress biomarker validation tool [51]. Risk groups were generated based on prognostic index (PI) calculations (higher values for higher risk), with PI being known as the linear component of the Cox model [52]. Two groups were split from the ordered PI according to SurvExpress optimization algorithm, with chosen split point corresponding to the lowest logrank *p*-value. KM-plotter [53] was used to investigate the impact on *THBS1* and *CD47* expression on patients’ overall and progression-free survival. Cutoff values for separating high and low gene expression groups were determined by the online algorithm.

### 4.9. Cell Lines

Human ovarian carcinoma cell lines A2780 and SK-OV-3 were obtained from Sigma-Aldrich (Saint-Quentin Fallavier, France) and the American Type Culture Collection (ATCC, Manassas, VA, USA), respectively. A2780 and SK-OV-3 cells were respectively maintained in RPMI 1640 medium containing 2 mM L-Alanyl-Glutamine (GlutaMAX^TM^-I, Gibco, Life Technologies, Saint-Aubin, France) and McCoy’s 5a medium modified (ATCC) supplemented with 10% heat-inactivated fetal bovine serum (FBS). ID8 mouse OC cell line was obtained from Dr. Katherine Roby (University of Kansas Medical Center) [41]. ID8 cells were maintained in high-glucose Dulbecco’s Modified Eagle’s Medium (#D6429, Sigma-Aldrich, Saint-Quentin Fallavier, France) supplemented with 4% FBS, 1% penicillin/streptomycin and insulin–transferrin–sodium selenite media supplement (ITS mix, #I1884, Sigma-Aldrich). Constitutive expression of TSP-1 and PD-L1 by ID8 cell line under unstimulated conditions was ensured by Western blot, using anti-TSP-1 (#ab1823; 1:1000 dilution) and anti-PD-L1 (#ab233482; 3 µg.mL^−1^) antibodies from Abcam. Cells were cultured in growth medium and split every 3 to 4 days by harvesting in trypsin. Authenticated cell lines by provider were stored at early passages (< 3) in liquid nitrogen, controlled to be mycoplasma-free, and were used in the experiments for no longer than 6 months.

### 4.10. Xenograft Models

Suspensions of A2780 or SK-OV-3 human ovarian carcinoma cells were s.c. inoculated (5 × 10^6^ cells in 200 µL per animal) into the left flank of BALB/c athymic nude mice. Tumor measurements (length, L; width, W) were performed twice a week, using a digital caliper, and total tumor volume was calculated according to V = 0.5 × L × W^2^. For randomization, mice were allocated to groups (*n* = 13–16 in each) based on similar mean and distribution of tumor volume on day 13. Systemic (i.p.) administrations of TAX2 peptide (10 mg · kg^−1^ mouse weight) or injectable saline (0.9% NaCl) were then performed three times a week for two weeks (A2780) or 7 weeks (SK-OV-3) starting two weeks after tumor cells inoculation. Body weight (BW) was monitored every 2 to 3 days, while clinical signs and behavior pattern were daily checked until study termination. Loss of 15% BW or tumor volume exceeding 1 cm^3^ were considered an indication for euthanasia, as well as severe tumor necrosis, as it was sometimes associated with loss of body fluid and/or infection.

### 4.11. Allograft Models

For subcutaneous studies, C57BL/6JRj mice (6 weeks old) were given a 300 µL subcutaneous injection of 5 × 10^6^ ID8 cells. When mean tumor volume reached 100–200 mm^3^, mice were randomized into the following treatment groups (*n* = 11 per group): vehicle (0.9% NaCl thrice weekly i.p.), TAX2 peptide (30 mg · kg^−1^ mouse weight, thrice weekly i.p.), anti-mouse PD-1 mAb (clone RMP1-14, #BE0146 from BioXCell, Euromedex, Souffelweyersheim, France; 200 µg, twice weekly i.p.) and combined TAX2/anti-PD-1 therapy (same schedule as each test item considered alone, i.e., TAX2 thrice weekly i.p. + anti-PD-1 twice weekly i.p.) over a 4-week treatment duration. For ethical and animal welfare reasons, we chose not to multiply the unnecessarily groups of animals and chose not to include groups with IgG control in the experimental design. However, we ensured that IgG isotype control (clone 2A3, #BE0089 from BioXCell, Euromedex, Souffelweyersheim, France) administration was comparable to NaCl. Tumor volume was measured every 2 to 3 days, while tumors were surgically extracted at study termination and then fixed in 4% paraformaldehyde for immunohistochemistry. For intraperitoneal studies, 5 × 10^6^ ID8 cells in a total volume of 400 µL PBS were transplanted to C57BL/6JRj mice (6 weeks old) via i.p. injections. Starting at day 14 after tumor inoculation, mice were treated as described above with either 0.9% NaCl (vehicle), TAX2 peptide, anti-PD-1 or TAX2/anti-PD-1 combination therapy (*n* = 11 per group). Treatment lasted 8 weeks, during which no signs of toxicity were observed in the drug-treated mice at either treatment exposure. Tumor progression was monitored by measuring abdominal distension (due to accumulation of malignant ascites fluid), using a digital caliper. At time of sacrifice, abdominal ascites fluid was extracted by using an 18-gauge needle. If no measurable ascites was present, peritoneal lavages were performed by injecting 8 mL PBS intraperitoneally and carefully extracting the fluid. Blood was also collected from the retro-orbital sinus, using heparin-coated capillary tube. Tumor loads were assessed by counting the number of tumor nodules on the parietal peritoneal surfaces and the visceral peritoneal surfaces of the intestine, liver, kidney and spleen. Peritoneal tumor masses were harvested and fixed with 4% paraformaldehyde for further analysis.

### 4.12. Histopathological Analysis

Histological analyses of paraffin-embedded tissues were performed on hematoxylin, eosin and saffron (HES)-stained 3 μm–thick sections that were prepared by using routine histological methods. Anti-CD31 (#ab182981, Abcam, Cambridge, UK; 1:1000), anti-Lyve-1 (#ab33682, Abcam, Cambridge, UK; 1:200), anti-CD4 (#ab183685, Abcam; 1:2000), anti-CD8 (#ab203035, Abcam; 1:250), anti-CD163 (#ab182422, Abcam; 1:500) and anti-TSP-1 (LS-C402945, LifeSpan Biosciences Inc.; 1:100) antibodies were used to perform immunostaining together with biotin-labeled secondary antibodies and streptavidin-HRP DAB (3,3’-diaminobenzidine) detection system (#64261, Abcam, Cambridge, UK), and then followed by hematoxylin/eosin counterstain (#RE7107, Leica Biosystems, Danaher Corporation, Washington, WA, USA). Negative controls were performed by omitting the primary antibody. The number of positive cells for each marker, as well as vessel density, was determined by an experienced pathologist who was blinded to the group’s treatment during the whole analysis procedure in 10 consecutive high-power fields (×400; 0.283 mm^2^) in the area with the highest density of staining. Moreover, automated quantitative image analyses across the whole s.c. allograft sections were performed, using ImageJ software binarization and thresholding tools.

### 4.13. Flow Cytometry Analyses (FCM) of Ascites

Ascites samples from the orthotopic ID8 OC model were spun at 300 g for 10 min to separate cellular and acellular fractions. Ascites cell pellets were incubated for 5 min with 5 mL of red cells lysis buffer (Versalyse lysing solution; #A09777, Beckman Coulter, Villepinte, France) and then washed with PBS. Pelleted cells were suspended in freezing medium (90% FBS/10% DMSO) and kept in liquid nitrogen for subsequent analysis. After thawing, cells were re-suspended in RPMI 1640 medium containing 10% FBS and 0.5% C_3_H_3_NaO_3_. Viable cells were then gated based on forward and side scatter profiles, and equal numbers of viable ascites cells were phenotyped, using the following fluorescent-labeled antibodies: CD3e-FITC (#11-0031-82, Thermo Fisher Scientific), CD45R-APC (#17-0452-82, Thermo Fisher Scientific), CD4-PE (#12-0041-82, Thermo Fisher Scientific) and CD8a-PerCP-Cyanine5.5 (#45-0081-82, Thermo Fisher Scientific) for 20 min, at room temperature. After washing with DPBS (#D8537, Sigma-Aldrich), stained samples were run on a BD Accuri™ C6 cytometer (Beckman Coulter) followed by gating, data analysis and visualization in FlowJo^TM^ (FlowJo LLC, Ashland, OR, USA) software.

### 4.14. Washed Platelets FCM Analysis

Washed platelets were freshly prepared as described elsewhere [54] from a pool of 4 mouse blood samples by treatment group (ID8 allograft i.p. model). Then, the putative impact of TAX2 peptide and/or anti-PD-1 treatment on platelet function was investigated by detection of activation in both resting and stimulated platelets. To promote activation, platelets were incubated with 3.33 U.mL^−1^ thrombin (#D8537, Sigma-Aldrich) for 5 min, at room temperature. Platelets were probed with both a (FITC)-conjugated monoclonal anti-CD41 antibody (clone MWReg30; #553848, BD Pharmingen) and a (PE)-conjugated monoclonal anti-CD62P antibody (clone Wug.E9; #M130-2, Emfret Analytics) for 10 min, at room temperature, in the dark. IgG1-PE (clone R3-34; #P190_2, Emfret Analytics) and IgG1-FITC (clone R3-34; #553924, BD Pharmingen) were employed as isotype controls. Platelet isolation was performed by gating in forward and side scatter, as well as relying on CD41 positivity, while CD62P (P-selectin) membrane exposure reflects platelet activation.

### 4.15. Inflammatory Cytokines Analyses

The non-cellular fraction from ascites samples (ID8 allograft i.p. model) was passed through a 0.45 µm filter, and then IFN-γ production was assessed by using a specific ELISA kit (#EA-4003, Signosis Inc., Santa Clara, CA, USA), according to the manufacturer’s protocol.

### 4.16. Statistical Analyses

GraphPad Prism 6.0c software (GraphPad Software Inc., San Diego, CA, USA) was used for all statistical analyses. Significance for Kaplan–Meier overall and progression-free survival analyses were assessed by logrank test to compare the survival distributions of low vs. high *THBS1*-expressing and *CD47*-expressing groups. Spearman’s *r* coefficient was calculated to estimate correlation between cell positivity for CD4 expression and change in allograft tumor volume. Comparison between two groups was performed by using Student’s *t*-test, and histograms display data as mean ± SEM. For in vivo experiments, groups were compared by using the non-parametric Mann–Whitney U test for unpaired samples. Multiple comparisons were performed with one-way analysis of variance (ANOVA), followed by Dunnett’s post hoc test. Two-sided *p*-values < 0.05 (*), <0.01 (**) or <0.001 (***) are indicated when statistical significance is reached.

## 5. Conclusions

Altogether, these data demonstrate that TAX2 peptide is a very promising candidate for further exploration as a novel cancer immunotherapeutic agent, especially for ovarian carcinoma. While IO + bevacizumab combinations are becoming standard treatments for several solid tumors, TAX2 may favorably come on top of one or the other thanks to its duality of action (i.e., anti-angiogenic and immunostimulatory). Indeed, we do believe that TAX2 is unique because it targets distinct components of the ovarian microenvironment at the same time with a single molecule. It is therefore a modulator of tumor-tolerant microenvironment, and not just another checkpoint inhibitor. Future work will focus on further documenting TAX2 immune mode of action in relevant animal models, while optimizing the best combination therapies with other IO products/targeted drugs or radiotherapy.

## Data Availability

No new data were created or analyzed in this study. Data sharing is not applicable to this article.

## Supplementary Materials

The following are available online at https://www.mdpi.com/article/10.3390/cancers13195019/s1. Figure S1: Western blot protein analyses of ID8 cells (whole membranes). Figure S2: A benchmark toxicity experiment of TAX2 peptide vs. anti-CD47 mAb. Figure S3: Flow cytometry analysis of the activatability of platelets isolated from ID8 tumor-bearing mice having received chronic TAX2 or combination therapy. Table S1: THBS1 and CD47 gene expression analysis: raw data from Oncomine platform analysis (normal ovary vs. ovarian carcinoma).

## Abbreviations

DLS, dynamic light scattering; ECM, extracellular matrix; OC, ovarian cancer; PARP, poly ADP ribose polymerase; SIRPα, signal regulatory protein alpha; SPR, surface plasmon resonance; TCGA, the cancer genome atlas; TiL, tumor-infiltrating lymphocyte; TSP-1, thrombospondin-1.

## References

[1] Jeanne A., Schneider C., Martiny L., Dedieu S. (2015). Original Insights on Thrombospondin-1-Related Antireceptor Strategies in Cancer. Front. Pharmacol..

[2] Sun S., Dong H., Yan T., Li J., Liu B., Shao P., Li J., Liang C. (2020). Role of TSP-1 as Prognostic Marker in Various Cancers: A Systematic Review and Meta-Analysis. BMC Med. Genet..

[3] Kaur S., Bronson S.M., Pal-Nath D., Miller T.W., Soto-Pantoja D.R., Roberts D.D. (2021). Functions of Thrombospondin-1 in the Tumor Microenvironment. Int. J. Mol. Sci..

[4] Resovi A., Pinessi D., Chiorino G., Taraboletti G. (2014). Current Understanding of the Thrombospondin-1 Interactome. Matrix Biol. J. Int. Soc. Matrix Biol..

[5] Huang T., Sun L., Yuan X., Qiu H. (2017). Thrombospondin-1 Is a Multifaceted Player in Tumor Progression. Oncotarget.

[6] Kazerounian S., Yee K.O., Lawler J. (2008). Thrombospondins in Cancer. Cell. Mol. Life Sci. CMLS.

[7] Sick E., Jeanne A., Schneider C., Dedieu S., Takeda K., Martiny L. (2012). CD47 Update: A Multifaceted Actor in the Tumour Microenvironment of Potential Therapeutic Interest. Br. J. Pharmacol..

[8] Maxhimer J.B., Soto-Pantoja D.R., Ridnour L.A., Shih H.B., Degraff W.G., Tsokos M., Wink D.A., Isenberg J.S., Roberts D.D. (2009). Radioprotection in Normal Tissue and Delayed Tumor Growth by Blockade of CD47 Signaling. Sci. Transl. Med..

[9] Miller T.W., Kaur S., Ivins-O’Keefe K., Roberts D.D. (2013). Thrombospondin-1 Is a CD47-Dependent Endogenous Inhibitor of Hydrogen Sulfide Signaling in T Cell Activation. Matrix Biol. J. Int. Soc. Matrix Biol..

[10] Kaur S., Schwartz A.L., Miller T.W., Roberts D.D. (2015). CD47-Dependent Regulation of H_2_S Biosynthesis and Signaling in T Cells. Methods Enzymol..

[11] Kaur S., Roberts D.D. (2016). Divergent Modulation of Normal and Neoplastic Stem Cells by Thrombospondin-1 and CD47 Signaling. Int. J. Biochem. Cell Biol..

[12] Stein E.V., Miller T.W., Ivins-O’Keefe K., Kaur S., Roberts D.D. (2016). Secreted Thrombospondin-1 Regulates Macrophage Interleukin-1β Production and Activation through CD47. Sci. Rep..

[13] Weiskopf K. (2017). Cancer Immunotherapy Targeting the CD47/SIRPα Axis. Eur. J. Cancer Oxf. Engl. 1990.

[14] Kojima Y., Volkmer J.-P., McKenna K., Civelek M., Lusis A.J., Miller C.L., Direnzo D., Nanda V., Ye J., Connolly A.J. (2016). CD47-Blocking Antibodies Restore Phagocytosis and Prevent Atherosclerosis. Nature.

[15] Huang Y., Ma Y., Gao P., Yao Z. (2017). Targeting CD47, the Achievements and Concerns of Current Studies on Cancer Immunotherapy. J. Thorac. Dis..

[16] Sikic B.I., Lakhani N., Patnaik A., Shah S.A., Chandana S.R., Rasco D., Colevas A.D., O’Rourke T., Narayanan S., Papadopoulos K. (2019). First-in-Human, First-in-Class Phase I Trial of the Anti-CD47 Antibody Hu5F9-G4 in Patients With Advanced Cancers. J. Clin. Oncol. Off. J. Am. Soc. Clin. Oncol..

[17] Anderson K.L., Snyder K.M., Ito D., Lins D.C., Mills L.J., Weiskopf K., Ring N.G., Ring A.M., Shimizu Y., Mescher M.F. (2019). Evolutionarily Conserved Resistance to Phagocytosis Observed in Melanoma Cells Is Insensitive to Upregulation of Pro-Phagocytic Signals and to CD47 Blockade. Melanoma Res..

[18] Guillon J., Petit C., Moreau M., Toutain B., Henry C., Roché H., Bonichon-Lamichhane N., Salmon J.P., Lemonnier J., Campone M. (2019). Regulation of Senescence Escape by TSP1 and CD47 Following Chemotherapy Treatment. Cell Death Dis..

[19] Qian X., Rothman V.L., Nicosia R.F., Tuszynski G.P. (2001). Expression of Thrombospondin-1 in Human Pancreatic Adenocarcinomas: Role in Matrix Metalloproteinase-9 Production. Pathol. Oncol. Res. POR.

[20] Pinessi D., Ostano P., Borsotti P., Bello E., Guffanti F., Bizzaro F., Frapolli R., Bani M.R., Chiorino G., Taraboletti G. (2015). Expression of Thrombospondin-1 by Tumor Cells in Patient-Derived Ovarian Carcinoma Xenografts. Connect. Tissue Res..

[21] Daubon T., Léon C., Clarke K., Andrique L., Salabert L., Darbo E., Pineau R., Guérit S., Maitre M., Dedieu S. (2018). Deciphering the Complex Role of Thrombospondin-1 in Glioblastoma Development. Nat. Commun..

[22] Jeanne A., Sick E., Devy J., Floquet N., Belloy N., Theret L., Boulagnon-Rombi C., Diebold M.-D., Dauchez M., Martiny L. (2015). Identification of TAX2 Peptide as a New Unpredicted Anti-Cancer Agent. Oncotarget.

[23] Jeanne A., Boulagnon-Rombi C., Devy J., Theret L., Fichel C., Bouland N., Diebold M.-D., Martiny L., Schneider C., Dedieu S. (2016). Matricellular TSP-1 as a Target of Interest for Impeding Melanoma Spreading: Towards a Therapeutic Use for TAX2 Peptide. Clin. Exp. Metastasis.

[24] Jeanne A., Untereiner V., Perreau C., Proult I., Gobinet C., Boulagnon-Rombi C., Terryn C., Martiny L., Brézillon S., Dedieu S. (2017). Lumican Delays Melanoma Growth in Mice and Drives Tumor Molecular Assembly as Well as Response to Matrix-Targeted TAX2 Therapeutic Peptide. Sci. Rep..

[25] Jeanne A., Martiny L., Dedieu S. (2016). Thrombospondin-Targeting TAX2 Peptide Impairs Tumor Growth in Preclinical Mouse Models of Childhood Neuroblastoma. Pediatr. Res..

[26] Jeanne A., Sarazin T., Charlé M., Kawecki C., Kauskot A., Hedtke T., Schmelzer C.E., Martiny L., Maurice P., Dedieu S. (2021). Towards the Therapeutic Use of Thrombospondin 1/CD47 Targeting TAX2 Peptide as an Antithrombotic Agent. Arterioscler. Thromb. Vasc. Biol..

[27] Anthis N.J., Clore G.M. (2013). Sequence-Specific Determination of Protein and Peptide Concentrations by Absorbance at 205 Nm. Protein Sci. Publ. Protein Soc..

[28] Floquet N., Dedieu S., Martiny L., Dauchez M., Perahia D. (2008). Human Thrombospondin’s (TSP-1) C-Terminal Domain Opens to Interact with the CD-47 Receptor: A Molecular Modeling Study. Arch. Biochem. Biophys..

[29] Kodama J., Hashimoto I., Seki N., Hongo A., Yoshinouchi M., Okuda H., Kudo T. (2001). Thrombospondin-1 and -2 Messenger RNA Expression in Epithelial Ovarian Tumor. Anticancer Res..

[30] Uhlén M., Fagerberg L., Hallström B.M., Lindskog C., Oksvold P., Mardinoglu A., Sivertsson Å., Kampf C., Sjöstedt E., Asplund A. (2015). Proteomics. Tissue-Based Map of the Human Proteome. Science.

[31] Wang C.-L., Lin M.-J., Hsu C.-Y., Lin H.-Y., Tsai H.-P., Long C.-Y., Tsai E.-M., Hsieh T.-H., Wu C.-H. (2019). CD47 Promotes Cell Growth and Motility in Epithelial Ovarian Cancer. Biomed. Pharmacother. Biomedecine Pharmacother..

[32] Soto-Pantoja D.R., Terabe M., Ghosh A., Ridnour L.A., DeGraff W.G., Wink D.A., Berzofsky J.A., Roberts D.D. (2014). CD47 in the Tumor Microenvironment Limits Cooperation between Antitumor T-Cell Immunity and Radiotherapy. Cancer Res..

[33] Li H., Wang D., Zhang H., Kirmani K., Zhao Z., Steinmetz R., Xu Y. (2009). Lysophosphatidic Acid Stimulates Cell Migration, Invasion, and Colony Formation as Well as Tumorigenesis/Metastasis of Mouse Ovarian Cancer in Immunocompetent Mice. Mol. Cancer Ther..

[34] Li C., Course M.M., McNeish I.A., Drescher C.W., Valdmanis P.N., Lieber A. (2020). Prophylactic in Vivo Hematopoietic Stem Cell Gene Therapy with an Immune Checkpoint Inhibitor Reverses Tumor Growth in Syngeneic Mouse Tumor Models. Cancer Res..

[35] Garnier L., Gkountidi A.-O., Hugues S. (2019). Tumor-Associated Lymphatic Vessel Features and Immunomodulatory Functions. Front. Immunol..

[36] Brandacher G., Winkler C., Schroecksnadel K., Margreiter R., Fuchs D. (2006). Antitumoral Activity of Interferon-Gamma Involved in Impaired Immune Function in Cancer Patients. Curr. Drug Metab..

[37] Zeng Y., Li B., Liang Y., Reeves P.M., Qu X., Ran C., Liu Q., Callahan M.V., Sluder A.E., Gelfand J.A. (2019). Dual Blockade of CXCL12-CXCR4 and PD-1-PD-L1 Pathways Prolongs Survival of Ovarian Tumor-Bearing Mice by Prevention of Immunosuppression in the Tumor Microenvironment. FASEB J. Off. Publ. Fed. Am. Soc. Exp. Biol..

[38] Zhang Q.-F., Li J., Jiang K., Wang R., Ge J.-L., Yang H., Liu S.-J., Jia L.-T., Wang L., Chen B.-L. (2020). CDK4/6 Inhibition Promotes Immune Infiltration in Ovarian Cancer and Synergizes with PD-1 Blockade in a B Cell-Dependent Manner. Theranostics.

[39] Crusz S.M., Miller R.E. (2020). Targeted Therapies in Gynaecological Cancers. Histopathology.

[40] Weng T.-Y., Huang S.-S., Yen M.-C., Lin C.-C., Chen Y.-L., Lin C.-M., Chen W.-C., Wang C.-Y., Chang J.-Y., Lai M.-D. (2014). A Novel Cancer Therapeutic Using Thrombospondin 1 in Dendritic Cells. Mol. Ther..

[41] Roby K.F., Taylor C.C., Sweetwood J.P., Cheng Y., Pace J.L., Tawfik O., Persons D.L., Smith P.G., Terranova P.F. (2000). Development of a Syngeneic Mouse Model for Events Related to Ovarian Cancer. Carcinogenesis.

[42] Yu D.L., Stegelmeier A.A., Chow N., Rghei A.D., Matuszewska K., Lawler J., Bridle B.W., Petrik J.J., Wootton S.K. (2020). AAV-Mediated Expression of 3TSR Inhibits Tumor and Metastatic Lesion Development and Extends Survival in a Murine Model of Epithelial Ovarian Carcinoma. Cancer Gene Ther..

[43] Behrendt R., White P., Offer J. (2016). Advances in Fmoc Solid-phase Peptide Synthesis. J. Pept. Sci..

[44] Hoofnagle A.N., Whiteaker J.R., Carr S.A., Kuhn E., Liu T., Massoni S.A., Thomas S.N., Townsend R.R., Zimmerman L.J., Boja E. (2016). Recommendations for the Generation, Quantification, Storage, and Handling of Peptides Used for Mass Spectrometry-Based Assays. Clin. Chem..

[45] Jenssen H., Aspmo S.I. (2008). Serum Stability of Peptides. Methods Mol. Biol. Clifton N.J..

[46] Di L., Kerns E.H., Hong Y., Chen H. (2005). Development and Application of High Throughput Plasma Stability Assay for Drug Discovery. Int. J. Pharm..

[47] Lipschultz C.A., Li Y., Smith-Gill S. (2000). Experimental Design for Analysis of Complex Kinetics Using Surface Plasmon Resonance. Methods San Diego Calif.

[48] Rhodes D.R., Kalyana-Sundaram S., Mahavisno V., Varambally R., Yu J., Briggs B.B., Barrette T.R., Anstet M.J., Kincead-Beal C., Kulkarni P. (2007). Oncomine 3.0: Genes, Pathways, and Networks in a Collection of 18,000 Cancer Gene Expression Profiles. Neoplasia N.Y.N..

[49] Cerami E., Gao J., Dogrusoz U., Gross B.E., Sumer S.O., Aksoy B.A., Jacobsen A., Byrne C.J., Heuer M.L., Larsson E. (2012). The CBio Cancer Genomics Portal: An Open Platform for Exploring Multidimensional Cancer Genomics Data. Cancer Discov..

[50] Gao J., Aksoy B.A., Dogrusoz U., Dresdner G., Gross B., Sumer S.O., Sun Y., Jacobsen A., Sinha R., Larsson E. (2013). Integrative Analysis of Complex Cancer Genomics and Clinical Profiles Using the CBioPortal. Sci. Signal..

[51] Aguirre-Gamboa R., Gomez-Rueda H., Martínez-Ledesma E., Martínez-Torteya A., Chacolla-Huaringa R., Rodriguez-Barrientos A., Tamez-Peña J.G., Treviño V. (2013). SurvExpress: An Online Biomarker Validation Tool and Database for Cancer Gene Expression Data Using Survival Analysis. PLoS ONE.

[52] Collett D. (2014). Modelling Survival Data in Medical Research.

[53] Nagy Á., Lánczky A., Menyhárt O., Győrffy B. (2018). Validation of MiRNA Prognostic Power in Hepatocellular Carcinoma Using Expression Data of Independent Datasets. Sci. Rep..

[54] Kawecki C., Hézard N., Bocquet O., Poitevin G., Rabenoelina F., Kauskot A., Duca L., Blaise S., Romier B., Martiny L. (2014). Elastin-Derived Peptides Are New Regulators of Thrombosis. Arterioscler. Thromb. Vasc. Biol..

