# Supplementary material for: Targeting Ovarian Carcinoma with TSP-1:CD47 Antagonist TAX2 Activates Anti-Tumor Immunity
Source: Cancers (Basel). 2021 Oct 7;13(19):5019. doi: 10.3390/cancers13195019 (PMC8508526; doi:10.3390/cancers13195019)
Supplement: Supplementary file 1 [file cancers-13-05019-s001.zip › cancers-1404093-supplementary.pdf]

Supplementary Materials: Targeting ovarian carcinoma with TSP-1:CD47 antagonist TAX2 activates anti-tumor immunity

Albin Jeanne, Thomas Sarazin, Magalie Charlé, Catherine Moali, Caroline Fichel, Camille Boulagnon-Rombi, Maïté Callewaert, Marie-Christine Andry, Eric Diesis, Frédéric Delolme, Damien Rioult and Stéphane Dedieu

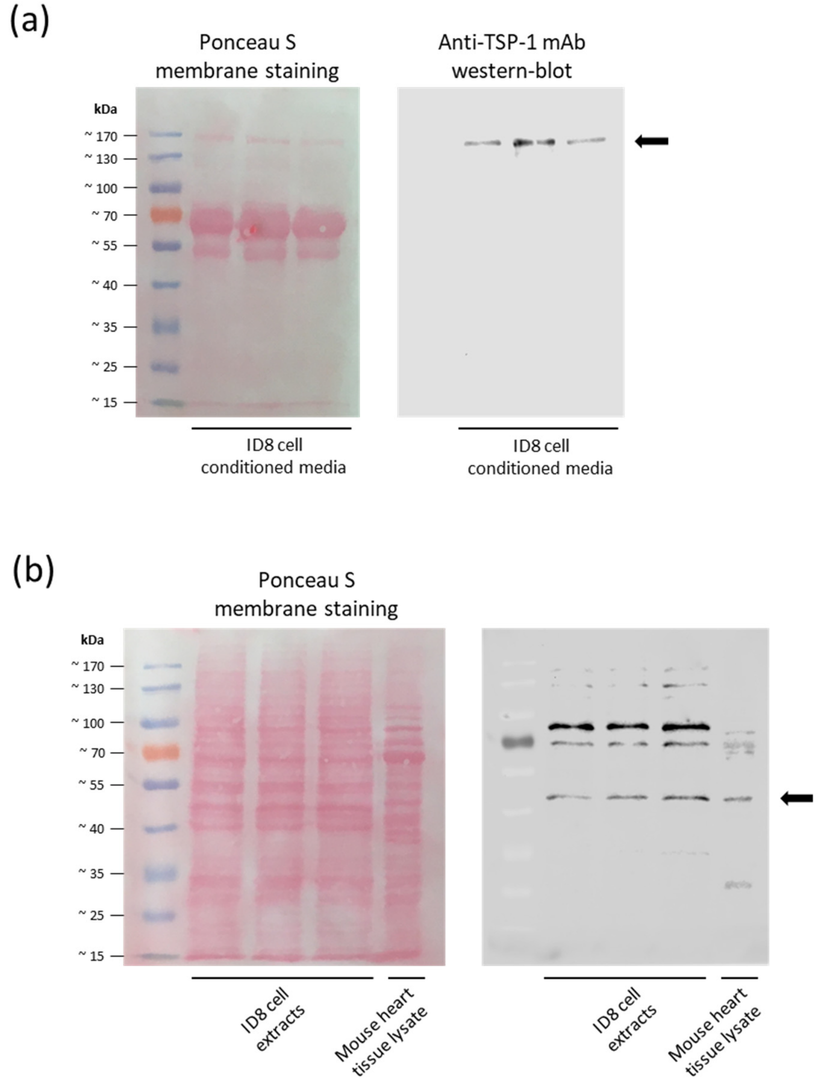

Figure S1. Western-blot protein analyses of ID8 cells (whole membranes). (a) 40 µg proteins from ID8 cell conditioned medium (24h incubation in RPMI1640 w/o FBS) were analyzed through SDS-page separation followed by Ponceau S red staining (left) and anti-TSP-1 (#ab1823; 1:1000 dilution) western-blot detection (right). (b) 40 µg proteins from ID8 cell extracts or mouse heart tissue lysate (positive control) were analyzed through SDS-page separation followed by Ponceau S red staining (left) and anti-PD-L1 (#ab233482; 3 µg.ml^{-1}) western-blot detection (right). Whole stained membranes and blots are shown, while arrows indicate bands of interest.

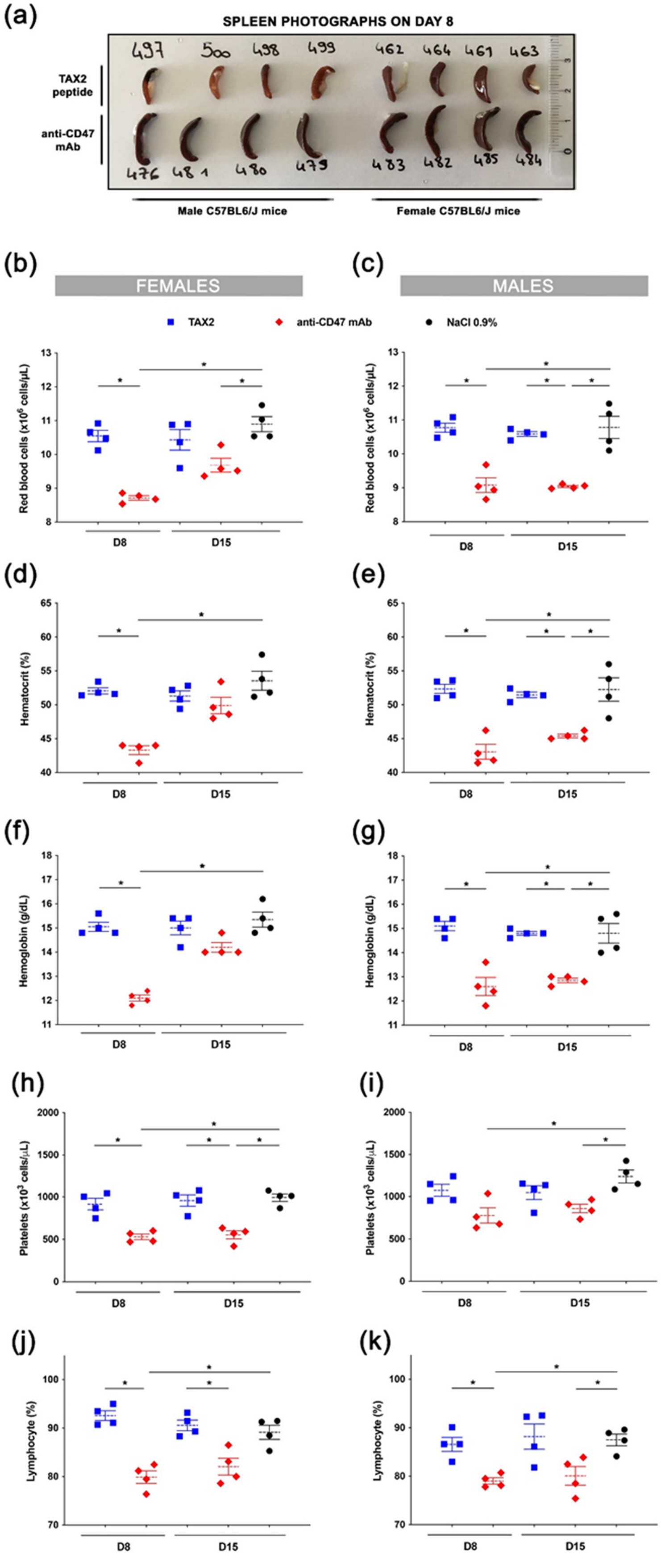
(a)
SPLEEN PHOTOGRAPHS ON DAY 8
TAX2 peptide
anti-CD47 mAb
497 500 498 499 462 464 461 463
476 481 480 479 483 482 485 484
Male C57BL6/J mice
Female C57BL6/J mice
(b)
FEMALES
(c)
MALES
TAX2
anti-CD47 mAb
NaCl 0.9%
Red blood cells (x10^6 cells/µL)
(d)
(e)
Hematocrit (%)
(f)
(g)
Hemoglobin (g/dL)
(h)
(i)
Platelets (x10^3 cells/µL)
(j)
(k)
Lymphocyte (%)
D8
D15

Figure S2. A benchmark toxicity experiment of TAX2 peptide vs. anti-CD47 mAb. 8 weeks old female and male C57BL/6JRj mice (n = 4 per group) received i.p. injections of TAX2 peptide (30 mg.kg-1 mouse weight, blue squares) or anti-CD47 mAb (200 µg/mouse/injection, red diamonds), administered either QD×7 or QD×14. Normal saline injections (0.9% NaCl, black circles) were also performed QD×14 to serve as internal reference. The day following the last dosing, blood sampling was performed from the retro-orbital sinus and then mice were euthanized prior to gross necropsy evaluation. (a) Photographs of spleen isolated on day 8 are shown. (b-k) Scatter dot plots display results obtained after hematology analysis in both female (b, d, f, h, j) and male (c, e, g, i, k) groups, including red blood cells count (b, c), hematocrit (d, e), hemoglobin concentration (f, g), platelets count (h, i) as well as leukocyte count expressed as percentage (j, k). For each analysis, mean ± SEM are also indicated together with results from two-tailed Mann-Whitney U test statistical analyses (* $p < 0.05$).

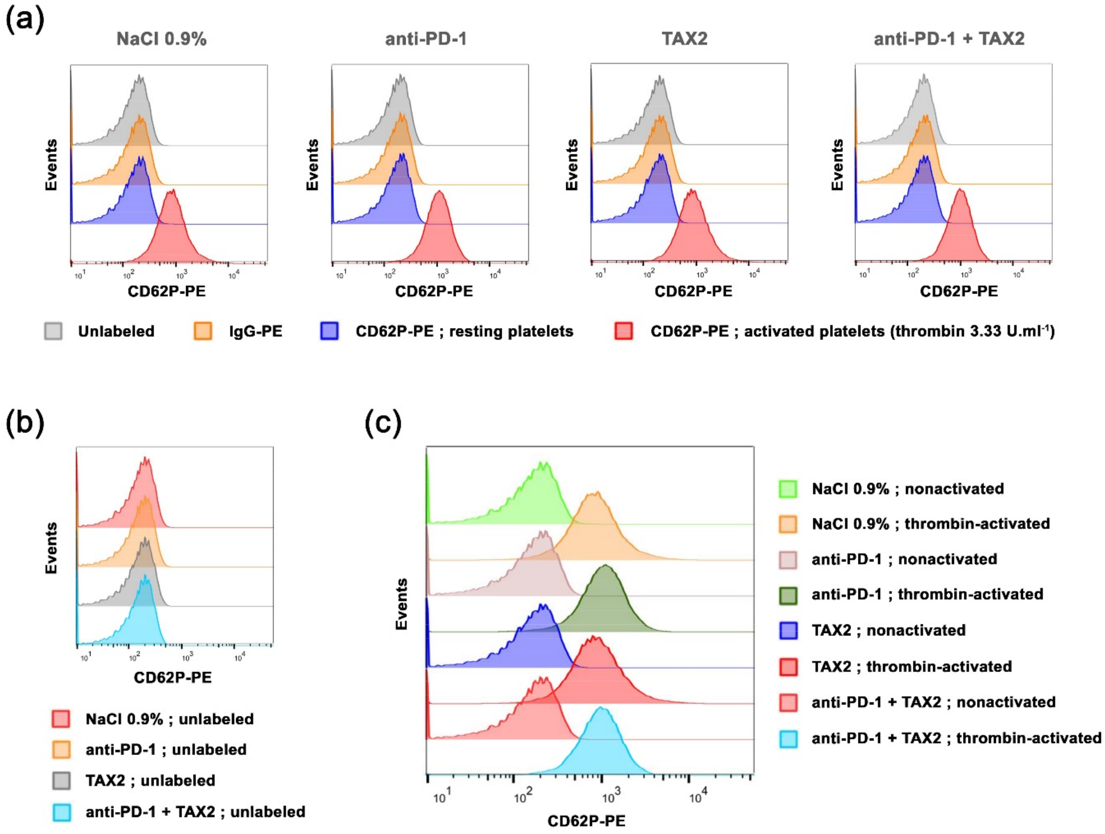

Figure S3. Flow cytometry analysis of the activatability of platelets isolated from ID8 tumor-bearing mice having received chronic TAX2 or combination therapy. Upon sacrifice, blood was collected from ID8 s.c. tumor bearing mice and then washed platelets were freshly prepared from a pool of 4 mouse samples by treatment group. The putative impact of treatments on platelet function was then investigated by flow cytometry. Platelet population was isolated based on anti-CD41 positivity, while platelet activation was detected using anti-CD62P (P-selectin). Activation was analyzed on both resting (i.e., unstimulated) and stimulated platelets using 3.33 U.ml-1 thrombin as an agonist. Whatever the administered treatment, no basal activation was reported, and platelets were still responding to thrombin-induced activation (a), while no change in the unlabeled control populations could be observed (b). Interestingly, the maximal amplitude (event number) as well as maximal activation (in terms of P-selectin exposure) were comparable between all groups (c).

Table S1. ***THBS1*** **and** ***CD47*** **gene expression analysis: raw data from Oncomine platform analysis (normal ovary vs. ovarian carcinoma).**

TCGA Ovarian Statistics (n = 594)				
Platform: Human Genome U1333A Array				
	THBS1 expression (reporter: 201110_s_at probe) log2 median-centered intensity		*CD47* expression (reporter: 213857_s_at probe) log2 median-centered intensity	
	Normal ovary	Ovarian carcinoma	Normal ovary	Ovarian carcinoma
Maximum	2.875	5.975	5.976	7.907
90th percentile	2.875	4.417	5.976	7.132
75th percentile	2.216	3.734	5.946	6.806
Median	0.813	2.795	5.499	6.508
25th percentile	0.099	1.824	5.306	6.066
10th percentile	-1.166	1.039	4.983	5.686
Minimum	-1.166	-1.112	4.983	
